# In Vitro Enzymatic and Kinetic Studies, and In Silico Drug-Receptor Interactions, and Drug-Like Profiling of the 5-Styrylbenzamide Derivatives as Potential Cholinesterase and β-Secretase Inhibitors with Antioxidant Properties

**DOI:** 10.3390/antiox10050647

**Published:** 2021-04-22

**Authors:** Malose J. Mphahlele, Emmanuel N. Agbo, Garland K. More, Samantha Gildenhuys

**Affiliations:** 1 Department of Chemistry, College of Science, Engineering and Technology, University of South Africa, Private Bag X06, Florida 1710, South Africa; piruesbest@yahoo.com; 2 Department of Life & Consumer Sciences, College of Agriculture and Environmental Sciences, University of South Africa, Private Bag X06, Florida 1710, South Africa; moregk@unisa.ac.za (G.K.M.); gildes@unisa.ac.za (S.G.)

**Keywords:** 5-styrylbenzamides, cholinesterase, β-secretase, antioxidant, metal chelating, drug-receptor interactions, cytotoxicity

## Abstract

The 5-(styryl)anthranilamides were transformed into the corresponding 5-styryl-2-(*p*-tolylsulfonamido)benzamide derivatives. These 5-styrylbenzamide derivatives were evaluated through enzymatic assays in vitro for their capability to inhibit acetylcholinesterase (AChE), butyrylcholinesterase (BChE), and β-secretase (BACE-1) activities as well as for antioxidant potential. An in vitro cell-based antioxidant activity assay involving lipopolysaccharides (LPS)-induced reactive oxygen species (ROS) production revealed that compounds **2a** and **3b** have the capability of scavenging free radicals. The potential of the most active compound, 5-styrylbenzamide (**2a**), to bind copper (II) or zinc (II) ions has also been evaluated spectrophotometrically. Kinetic studies of the most active derivatives from each series against the AChE, BChE, and β-secretase activities have been performed. The experimental results are complemented with molecular docking studies into the active sites of these enzymes to predict the hypothetical protein–ligand binding modes. Their drug likeness properties have also been predicted.

## 1. Introduction

It is estimated that, if no preventative measures are made available against Alzheimer’s disease (AD), the number of the elderly suffering from this central nervous system (CNS) disorder will increase to 150 million by 2050 [1]. This age-related neurodegenerative disease is characterized by progressive and irreversible cognitive deterioration, loss of memory, and behavioral disturbances. Individuals suffering from this neurodegenerative disorder, on the other hand, require constant human and medical care, which create a heavy socio-economic burden on the health care system [2]. Continued research on AD has revealed that low levels of acetylcholine, formation of β-amyloid (Aβ) deposits, oxidative stress, metal imbalance, inflammation, and immune suppression are involved in the pathogenesis and progression of AD [3,4]. The brains of AD patients generally show abnormal accumulation of β-amyloid (Aβ) plaques and the deposition of neurofibrillary tangles associated with oxidative damage [5]. The antioxidant system loses effectiveness during the aging process and, as a result, oxidative stress leads to neuronal degeneration and cholinergic dysfunctions in the brain [6]. Oxidative stress is one of the earliest events in the progress of AD observed before the formation of AD-specific pathological Aβ plagues [6,7]. Excessive bio-metals (Cu^2+^, Fe^2+^, Zn^2+^ and Al^3+^) have been found within the Aβ deposits in AD brains and the interaction of these bio-metals with Aβ contributes to the production of reactive oxygen species (ROS) [8]. The most common therapeutic approach to counteract the effect of this multifactorial disease with interrelated factors is to raise the concentration of acetylcholine (ACh) through the inhibition of activities of acetylcholinesterase (AChE) and butyrylcholinesterase (BChE), which are responsible for the hydrolysis of ACh in the synaptic clefts. However, cholinesterase inhibitors only partly compensate the lost cognitive functions and have proven to be insufficient to slow down or to stop the neurodegenerative process itself [3,4]. It is envisioned that the treatment of AD would benefit from the use of multipotent drugs that target at least two or more key pathophysiological processes linked to AD, such as cholinesterases (AChE and/or BChE) and β-secretase activities as well oxidative stress [9,10].

**Figure 1:**
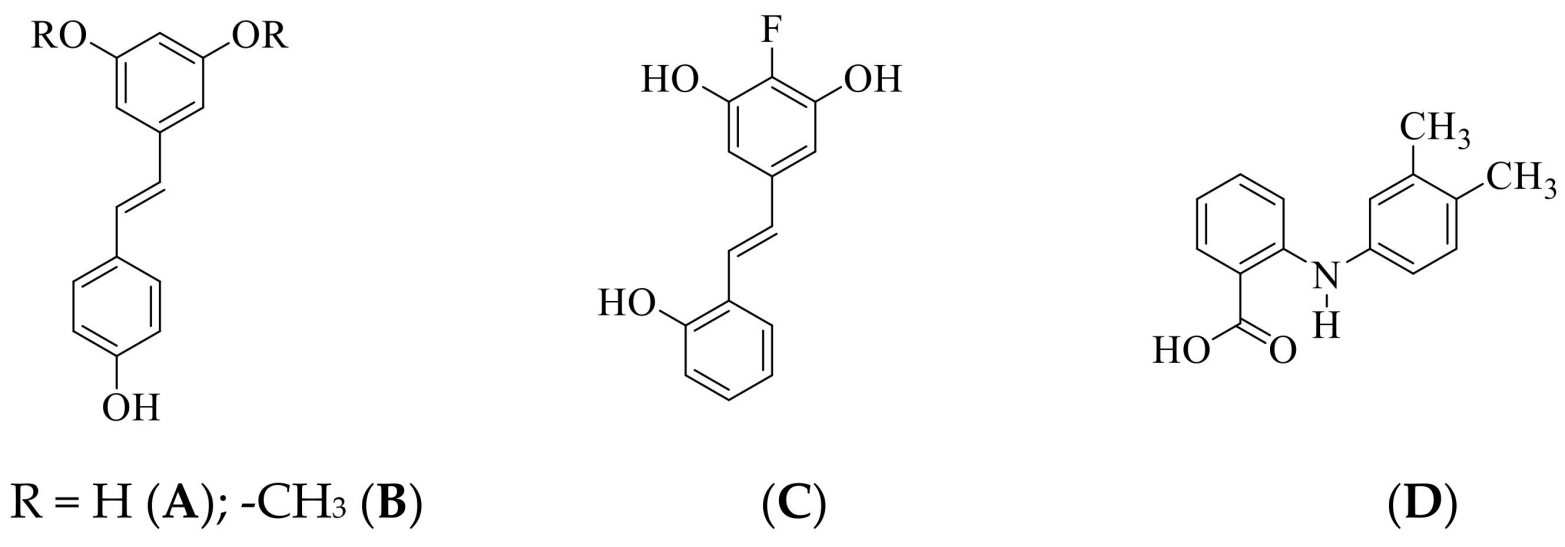
Structures of natural (**A**,**B**) and synthetic (**C**) stilbenes, and mefenamic acid (**D**).

The naturally occurring stilbene (1,2–diphenylethylene) derivatives, such as resveratrol (*trans*-3,4,5-trihydroxystilbene) (**A**) and pterostilbene (*trans*-3,5-dimethoxy-4-hydroxystilbene) (**B**), have been proven to be potent multiple molecular modulators for age-related diseases, including oxidative damage, inflammation, neurodegeneration, obesity, diabetes, and cardiovascular diseases [11,12,13,14]. The biological properties of stilbenes appear to be dependent on the electron-donating or electron-withdrawing nature of the functional groups at the 4 and 4׳ positions, respectively [15]. The synthetic derivatives of resveratrol derivatives have previously been evaluated for anticholinesterase activity against AChE and BChE, and one of the derivatives (**C)** substituted with a fluorine atom was found to be 94–fold more active and selective against BChE than against AChE [14]. Mefenamic acid (**D**), which is an anthranilic acid derivative shown in Figure 1, was found to exert neuroprotective effects and to improve cognitive impairment in vitro and in vivo in Alzheimer’s disease models [16]. The C-2 *N*-substituted anthranilamide derivatives as isoesteres of anthranilic acid have previously been prepared and evaluated for anticholinesterase properties [17]. The primary requirement for drugs that target the CNS is to be lipophilic and have smaller sizes (less than 500 Dalton) in order to pass through the blood–brain barrier (BBB) [18]. This membrane protects the brain from harmful chemicals and makes it difficult for drugs to penetrate or cross through to reach target cells and exert their effects. Anthranilamide derivatives have previously been found to inhibit neurotropic alphavirus replication, and to achieve measurable brain permeation in a pharmacokinetic mouse model [19]. Increased activity and membrane permeability were attributed to the presence of the lipophilic NH, and its propensity to participate in a six-membered intramolecular hydrogen bonding interaction with an oxygen atom of the *ortho* carbonyl group.

2-Aminobenzamide and its *N*^2^-substituted derivatives contain both the carbonyl and amino groups bearing high electronegativity O and N atoms, and these amino acid analogues are capable of coordinating transition metals. 2-Aminobenzamide moiety, for example, is a zinc-binding group in the molecular structure of histone diacetylase (HDAC) inhibitors [20]. Moreover, an amide moiety gives the compound a neutral charge and has previously been found to improve the lipophilicity, BBB permeability, and antioxidant property of *N*-acetylcysteine amide (NACA) more so than its precursor, *N*-acetylcysteine (NAC) [21]. Its propensity for hydrogen bonding plays an important role in the spatial structure of proteins, nucleic acids, and biological membranes as well as in the interaction of bioactive compounds with receptors. We considered the anticholinesterase activities of stilbene and benzamide (anthranilamide) derivatives, and decided to link these scaffolds on the same molecular framework for further transformation of the intermediate 5-styrylbenzamides into the corresponding 5-styryl-2-(*p*-tolylsulfonamido)benzamide derivatives. Sulfonamide-based compounds exhibit inhibitory properties against various enzymes including carbonic anhydrase, cysteine protease, HIV protease, cyclooxygenase, acetylcholinesterase (AChE), and butyrylcholinesterase (BChE) [22]. This moiety is capable of establishing relatively strong electrostatic and hydrogen bonding interactions with a wide range of functional groups of the protein residues [23]. Moreover, *p*-toluenesulfonamide as an anticancer agent has been found to exhibit good lipophilicity [24]. The 5-styrylbenzamides and their 5-styryl-2-(*p*-tolylsulfonamido)benzamide derivatives were, in turn, evaluated for an inhibitory effect in vitro against cholinesterase (AChE and BChE) and β-secretase activities, and for antioxidant potential through the 2,2-diphenyl-1-picrylhydrazyl (DPPH) and nitric oxide (NO) radical scavenging assays. A cell-based antioxidant activity assay on the most active compounds involving lipopolysaccharide (LPS)-induced reactive oxygen species (ROS) production in the normal monkey kidney cells (Vero) and adeno-carcinomic human epithelial (A549) cells was performed. The potential of the most active compound from each series to bind copper (II) or zinc (II) ions has also been evaluated spectrophotometrically. Kinetic assays (in vitro) and molecular docking (in silico) were performed on the most active compound among the test compounds against these enzymes to determine plausible protein-drug interactions on a molecular level. The ADMET (absorption, distribution, metabolism, and excretion) properties of the most active compounds from each series have also been predicted using an in-silico method.

## 2. Materials and Methods

### 2.1. Instrumentation and Materials

The melting point values of the test compounds were recorded on a Thermocouple digital melting point apparatus (Mettler Toledo LLC, Columbus, OH, USA). The infrared (IR) spectra were recorded using the thin-film method on a Bruker VERTEX 70 FT-IR Spectrometer (Bruker Optics, Billerica, MA, USA) equipped with an ATR (diamond attenuated total reflectance) accessory. Merck kieselgel 60 (0.063–0.200 mm) (Merck KGaA, Frankfurt, Germany) was used as a stationary phase for column chromatography. The ^1^H-NMR and ^13^C-NMR spectra of the prepared compounds were obtained as deuterated dimethyl sulfoxide ((CD_3_)_2_SO) solutions using Agilent 500 MHz NMR spectrometer (Agilent Technologies, Oxford, UK) operating at 500 MHz and 125 MHz for ^1^H and ^13^C, respectively. The chemical shifts are quoted relative to tetramethylsilane (TMS) used as an internal reference standard (δ = 0.00 ppm) or a residual protonated solvent. The high-resolution mass spectra were recorded at an ionization potential of 70 eV using Micromass Autospec-TOF (double focusing high resolution) instrument (Waters Corp., Milford, MA, USA). The synthesis and analytical data for 2-amino-5-iodobenzamide (**1**) have been reported in our previous study [25]. Recombinant human AChE (BioLegend) and BChE (Cloud-Clone Corp.) were purchased via Biocom Africa (Pty) Ltd. (Centurion, Pretoria, South Africa).

### 2.2. Typical Procedure for the Synthesis of the 5-Styrylbenzamides ***2a**–**d***

A mixture of **1** (0.50 g, 1.91 mmol), PdCl_2_(PPh_3_)_2_ (0.07 g, 0.10 mmol), PCy_3_ (0.26 g, 0.19 mmol), and K_2_CO_3_ (0.30 g, 2.30 mmol) and phenylboronic acid (0.40 g, 2.30 mmol) in 3:1 DMF–water (*v*/*v*, 20 mL) in a two-necked round bottom flask equipped with a condenser and rubber septum was purged with nitrogen gas for 30 min. A balloon filled with nitrogen gas was connected to the top of the condenser, and the reaction mixture was stirred at 70 °C for 3 h. The mixture was quenched with ice-cold water, and the product was extracted into chloroform. The combined organic layers were washed with water, dried over anhydrous MgSO_4._, and the salt was filtered off. The solvent was evaporated under reduced pressure on a rotary evaporator. The residue was purified by column chromatography on silica gel using 2:1 toluene-ethyl acetate (*v*/*v*) mixture as an eluent. Compounds **2a**–**d** were prepared in this fashion.

(*E*)-2-Amino-5-styrylbenzamide (**2a**).

White solid (0.36 g, 80%), mp. 210–211 °C, ν_max_ (ATR) 682, 813, 1158, 1337, 1506, 1582, 1739, 3023, 3222, 3406 cm^−1^, ^1^H-NMR ((CD_3_)_2_SO) 6.69 (1H, d, *J =* 8.5 Hz, H-3), 6.79 (2H, s, -NH_2_), 7.00 (1H, d, *J*_trans_ = 16.5 Hz, H-α), 7.04 (1H, d, *J*_trans_ = 16.5 Hz, H-β), 7.13 (1H, br s, -NHC=O), 7.19 (1H, t, *J* = 7.5 Hz, Ph), 7.33 (2H, t, *J* = 7.5 Hz, Ph), 7.41 (1H, dd, *J* = 1.5 and 8.5 Hz, H-4), 7.48 (2H, d, *J* = 7.5 Hz, Ph), 7.79 (1H, d, *J* = 1.5 Hz, H-6), 7.83 (1H br s, -NHC=O), ^13^C-NMR ((CD_3_)_2_SO) 114.0, 117.1, 124.1, 124.2, 126.2, 126.2, 127.1, 127.8, 128.9, 129.2, 130.7, 138.2, 150.5, 171.6; HRMS (ES): *m*/*z* [M + H]^+^ calc for C_15_H_15_N_2_O: 239.1184; found 239.1185.

(*E*)-2-Amino-5-(4-fluorostyryl)benzamide (**2b**).

White solid (0.37 g, 75%), mp. 224–225 °C, ν_max_ (ATR) 682, 817, 1158, 1507, 1582, 1642, 1739, 3163, 3405 cm^−1^, ^1^H-NMR ((CD_3_)_2_SO) 6.69 (1H, d, *J =* 8.5 Hz, H-3), 6.75 (2H, s, -NH_2_), 6.96 (1H, d, *J*_trans_ = 16.5 Hz, H-α), 7.01 (1H, d, *J*_trans_ = 16.5 Hz, H-β), 7.13 (1H, br s, -NHC=O), 7.16 (2H, d, *J* = 8.7 Hz, H-2′,6′) 7.39 (1H, dd, *J* = 1.5 and 8.5 Hz, H-4), 7.51 (2H, dd, *J*_HF_ = 5.5 and *J*_HH_ = 8.5 Hz, H-3′,5′), 7.77 (1H, d, *J* = 1.5 Hz, H-6), 7.82 (1H br s, -NHC=O); ^13^C-NMR ((CD_3_)_2_SO) 114.0, 116.0 (d, ^2^*J*_CF_ = 20.9 Hz), 117.1, 123.1, 124.0, 127.7, 127.9 (d, ^2^*J*_CF_ = 7.6 Hz), 128.8, 130.7, 134.8 (d, ^3^*J*_CF_ = 2.9 Hz), 150.4, 161.6 (d, ^1^*J*_CF_ = 242.8 Hz), 171.6, HRMS (ES): *m*/*z* [M + H]^+^ calc for C_15_H_14_FN_2_O: 257.1090; found 257.1089.

(*E*)-2-Amino-5-(4-chlorostyryl)benzamide (**2c**).

White solid (0.38 g, 73%), mp. 240–241 °C, ν_max_ (ATR) 706, 813, 1158, 1506, 1582, 1643, 1739, 3161, 3313 cm^−1^, ^1^H-NMR ((CD_3_)_2_SO) 6.69 (1H, d, *J* = 8.5 Hz, H-3), 6.79 (2H, s, NH_2_), 6.99 (1H, d, *J*_trans_ = 16.5 Hz, H-α), 7.05 (1H, d, *J*_trans_ = 16.5 Hz, H-β), 7.13 (1H, br s, -NHC=O), 7.38 (2H, d, *J* = 8.5 Hz, H-2′,6′), 7.40 (1H, dd, *J* = 1.5 and 8.5 Hz, H-4), 7.50 (2H, d, *J* = 8.5 Hz, H-3′,5′), 7.78 (1H, d, *J* = 1.5 Hz, H-6), 7.90 (1H br s, -NHC=O), ^13^C-NMR ((CD_3_)_2_SO) 114.0, 117.1, 122.8, 123.8, 127.8, 127.9, 129.1, 129.9, 130.8, 131.3, 137.2, 150.6, 171.5; HRMS (ES): *m*/*z* [M + H]^+^ calc for C_15_H_14_ClN_2_O: 273.0795; found 273.0799.

(*E*)-2-Amino-5-(4-methoxystyryl)benzamide (**2d**).

White solid (0.36 g, 70%), mp. 214–216 °C, ν_max_ (ATR) 824, 958, 1023, 1229, 1508, 1571, 1624, 1739, 3192, 3472 cm^−1^, ^1^H-NMR ((CD_3_)_2_SO) 3.75 (3H, s, -OCH_3_), 6.67 (1H, d, *J* = 8.5 Hz, H-3), 6.70 (2H, s, NH_2_), 6.87 (1H, *J*_trans_ = 16.5 Hz, H-α), 6.91 (2H, d, *J* = 8.5 Hz, H-2′,6′), 6.95 (1H, d, *J*_trans_ = 16.5 Hz, H-β), 7.12 (1H, br s, -NH-C=O), 7.36 (1H, dd, *J* = 1.5 and 8.5 Hz, H-4), 7.41 (2H, d, *J* = 8.5 Hz, H-3′,5′), 7.74 (1H, d, *J* = 1.5 Hz, H-5), 7.82 (1H, br s, -NHC=O), ^13^C-NMR ((CD_3_)_2_SO) 55.6, 114.0, 114.6, 117.2, 124.0, 124.5, 126.7, 127.3, 127.4, 130.4, 130.9, 150.1, 158.8, 171.6, HRMS (ES): *m*/*z* [M + H]^+^ calc for C_16_H_17_N_2_O_2_: 269.1290; found 269.1289.

### 2.3. Typical Procedure for the Synthesis of the (E) 5-styryl-2-(p-tolylsulfonyl)benzamide ***3a**–**d***

*p*-Toluenesulfonyl chloride (1.2 equiv.) was added gradually to a stirred solution of **2** (1 equiv.) in pyridine (2 mL/mmol of **2**) at 0–5 °C. The mixture was allowed to warm to room temperature and stirred for 3 h at this temperature. The mixture was quenched with ice-cold water, and the precipitate was filtered and purified by silica gel column chromatography using 40% ethyl acetate-toluene mixture as an eluent. Compounds **3a**–**d** were prepared in this fashion.

(*E*)-5-Styryl-2-[[(4-methylphenyl)sulfonyl]amino]benzamide (**3a**).

White solid (0.23 g, 69%), mp. 174–175 °C, ν_max_ (ATR) 768, 1159, 1218, 1507, 1602, 1668, 1739, 2971, 3196, 3384 cm^−1^, ^1^H-NMR ((CD_3_)_2_SO) 2.07 (3H, s, -CH_3_), 7.10 (1H, d, *J*_trans_ = 16.5 Hz, H-α), 7.23 (1H, d, *J*_trans_ = 16.5 Hz, H-β), 7.25 (1H, d, *J* = 8.0 Hz, H-3), 7.35–7.37 (5H, m, Ph), 7.51 (2H, d, *J* = 6.5 Hz, H-3′,5′), 7.65 (2H, d, *J* = 8.0 Hz, H-2′,6′), 7.93 (1H, s, -NHC=O), 8.03 (1H, d, *J* = 1.2 Hz, H-6), 8.42 (1H, s, -NHC=O), 12.12 (1H, s, -NHS), ^13^C-NMR ((CD_3_)_2_SO) 21.4, 119.6, 119.8, 126.8 (2×C), 126.9, 127.3, 128.2, 129.3 (2×C), 130.3, 131.5, 132.5, 136.4, 137.3, 138.7, 144.3, 171.0, HRMS (ES): *m*/*z* [M + H]^+^ calc for C_22_H_21_N_2_O_3_S: 393.1273; found 393.1274.

(*E*)-5-(4-Fluorostyryl)-2-[[(4-methylphenyl)sulfonyl]amino]benzamide (**3b**).

White solid (0.24 g, 72%), mp. 198–199 °C, ν_max_ (ATR) 829, 1157, 1227, 1508, 1655, 1739, 2971, 3196, 3457 cm^−1^, ^1^H-NMR ((CD_3_)_2_SO) 2.30 (3H, s, -CH_3_), 7.02 (1H, d, *J*_trans_ = 16.5 Hz, H-α), 7.20 (2H, t, *J* = 9.0 Hz, H-2′,6′), 7.23 (1H, d, *J*_trans_ = 16.5 Hz, H-β), 7.32 (2H, d, *J* = 8.0 Hz, H-3″,5″), 7.51 (1H, d, *J* = 9.0 Hz, H-3), 7.57 (2H, dd, *J*_HF_ = 6.0 and *J*_HH_ = 9.0 Hz, H-3′,5′) 7.62 (1H, dd, *J* = 1.5 and 8.5 Hz, H-4), 7.65 (2H, d, *J* = 8.5 Hz, H-2″,6″), 7.94 (1H, br s, -NHC=O), 8.01 (1H, d, *J* = 2.0 Hz, H-6), 8.41 (1H, br s, -NHC=O), 12.11 (1H, s, -NH), ^13^C-NMR ((CD_3_)_2_SO) 21.4, 110.0, 116.0 (d, ^2^*J*_CF_ = 21.8 Hz), 119.7, 119.9, 126.9, 127.2, 127.3, 128.1, 128.7 (d, ^2^*J*_CF_ = 7.6 Hz), 130.3, 131.4, 132.5, 134.0 (d, ^3^*J*_CF_ = 3.8 Hz), 136.4, 138.7, 144.3, 162.3 (d, ^1^*J*_CF_ = 243.8 Hz), 171.0, HRMS (ES): *m*/*z* [M + H]^+^ calc for C_22_H_20_FN_2_O_3_S: 411.1179; found 411.1185.

(*E*)-5-(4-Chlorostyryl)-2-[[(4-methylphenyl)sulfonyl]amino]benzamide (**3c**).

White solid (0.21 g, 70%), mp. 224–225 °C, ν_max_ (ATR) 832, 1090, 1163, 1217, 1506, 1659, 1739, 3027, 3171, 3471 cm^−1^, ^1^H-NMR ((CD_3_)_2_SO) 2.31 (3H, s, -CH_3_), 7.12 (1H, d, *J*_trans_ = 16.5 Hz, H-α), 7.21 (1H, d, *J*_trans_ = 16.5 Hz, H-β), 7.33 (2H, d, *J* = 8.5 Hz, H-3″,5″), 7.42 (2H, d, *J* = 8.5 Hz, H-2″,6″), 7.51 (1H, d, *J* = 9.0 Hz, H-3), 7.54 (2H, d, 8.0 Hz, H-3′,5′), 7.62 (2H, d, 8.0 Hz, H-2,6), 7.64 (1H, t, *J* = 8.5 Hz, H-4), 7.94 (1H, br s, -NHC=O), 8.02 (1H, d, *J* = 2.0 Hz, H-6), 8.41 (1H, br s, -NHC=O), 12.11 (1H, s, -NH), ^13^C-NMR ((CD_3_)_2_SO) 21.4, 119.6, 119.9, 127.1, 127.2, 127.9, 128.2, 128.4, 129.3, 130.3, 131.6, 132.3, 132.5, 136.3, 136.4, 138.8, 144.3, 171.0, HRMS (ES): *m*/*z* [M + H]^+^ calc for C_22_H_20_ClN_2_O_3_S: 427.0883, found 427.0886.

(*E*)-5-(4-Methoxystyryl)-2-[[(4-methylphenyl)sulfonyl]amino]benzamide (**3d**).

White solid (0.21 g, 66%), mp. 174–175 °C, ν_max_ (ATR) 828, 1157, 1510, 1657, 1738, 2927, 3193, 3444 cm^−1^, ^1^H-NMR ((CD_3_)_2_SO) 2.30 (3H, s, -CH_3_), 3.75 (3H, s, -OCH_3_), 6.92 (2H, d, 8.0 Hz, H-3′5′), 6.94 (1H, d, *J*_trans_ = 16.5 Hz, H-α), 7.14 (1H, d, *J*_trans_ = 16.5 Hz, H-β), 7.32 (2H, d, *J* = 8.5 Hz, H-3″,5″), 7.46 (1H, d, *J* = 8.0 Hz, H-3), 7.50 (2H, d, *J* = 8.5 Hz, H-2′,6′), 7.59 (1H, dd, *J* = 1.5 and 8.5 Hz, H-4), 7.63 (2H, d, *J* = 8.5 Hz, H-2″,6″), 7.90 (1H, br s, -NHC=O), 7.96 (1H, d, *J* = 2.0 Hz, H-6), 8.39 (1H, br s, -NHC=O), 12.06 (1H, s, -NH), ^13^C-NMR ((CD_3_)_2_SO) 21.4, 55.6, 114.7, 119.8, 119.9, 124.9, 126.9, 127.2, 128.1, 128.9, 129.9, 130.3, 131.1, 133.0, 136.4, 138.2, 144.3, 159.5, 171.1, HRMS (ES): *m*/*z* [M + H]^+^ calc for C_23_H_23_N_2_O_4_S: 422.1379; found 422.1382.

### 2.4. Cholinesterase Inhibition Assays of ***2a**–**d*** and ***3a**–**d***

AChE and BChE were used to determine cholinesterase activities spectrophotometrically using acetylcholine iodide and butyrylcholine iodide as substrates, respectively, following a modification of the Ellman’s method, as described in our previous study [26], and the reactions were performed in triplicate at 37 °C in a 96-well plate. The stock solutions (200 μM) of both the test compounds and reference standard (donepezil) were prepared in DMSO, and further diluted to 5, 10, 25, 50, and 100 μM using Tris buffer (50 mM, pH 7.7). A multi-channel pipette was used for the addition of reagents to the wells.

#### 2.4.1. AChE Inhibition Assay of **2a**–**d** and **3a**–**d**

To each well of a 96-well plate, 8.0 μL solution of the test compound, 2.0 μL of AChE (0.04 mg/mL), and 70 μL of Tris buffer (C_4_H_11_NO_3_, 50 mM, pH 7.7) were added sequentially. The assay mixture was pre-incubated for 30 min at room temperature, which was followed by the addition of 10 μL of 5,5′-dithiobis-(2-nitrobenzoic acid) (DTNB, 3 mM in Tris buffer, 50 mM, pH 7.7) and 10 μL of acetylcholine iodide (AChI, 5 mM in tris buffer, 50 mM, pH 7.7) to each well to initiate the reaction. Five different absorbance readings were recorded for each triplicate run at a wavelength of 412 nm using a Varioskan flash spectrophotometer plate reader (Thermo Scientific, Waltham, MA, USA). The average values obtained from the absorbance readings were used to determine the IC_50_ and standard deviation values using the Graph Pad Prism (version 5.0, GraphPad software Inc., San Diego, CA, USA).

#### 2.4.2. BChE Inhibition Assay of **2a**–**d** and **3a**–**d**

A total of 8.0 μL of the test compound and 2.0 μL of BChE (0.02 mg/mL) was added to each of the designated wells of the 96-well plate, and the mixtures were incubated at room temperature for 30 min. Tris buffer (70 μL) was added to each well containing the reaction mixture and incubation was continued for a further 10 min at this temperature, which was followed by the addition of 10 μL solution of 5,5′-dithiobis-(2-nitrobenzoic acid) (DTNB) (3 mM in Tris buffer, 50 mM, pH 7.7) and butyrylcholine iodide (5 mM in Tris buffer, 50 mM, pH 7.7) to each well to initiate the reaction. Five different absorbance readings were recorded for each triplicate run at a wavelength of 412 nm using a Varioskan flash spectrophotometer plate reader (Thermo Scientific, Waltham, MA, USA). The IC_50_ and standard deviation values using the Graph Pad Prism.

### 2.5. β-Secretase (BACE-1) Inhibition Assay of ***2a**–**d*** and ***3a**–**d***

The β-secretase inhibitory activities of the test compounds were assayed in triplicates in 96-well plates using a recombinant baculovirus-expressed β-secretase and a specific substrate (Rh-EVNLDAEFK-Quencher), according to the manufacturer’s (Thermo Fisher Scientific Corporation, Johannesburg, Gauteng, South Africa) instructions, as described in our previous study [26]. The stock solutions (200 μM) of both the test compounds and reference standard (quercetin) were prepared in DMSO, and further diluted to 5, 10, 25, 50, and 100 μM using Tris buffer (50 mM, pH 7.7). In total, 10 μL of the test compounds and quercetin were added to the designated wells of the 96-well plate, respectively. Afterward, 10 μL of substrate (75 μM in 50 mM ammonium bicarbonate diluted with the assay buffer to 7.6 nM) were added to each well. To each well, 2.0 μL human recombinant β-secretase (1.0 U/mL) was added to start the reaction and the reaction mixture was incubated for 60 min at room temperature. Thereafter, 10 μL of the BACE-1 stop buffer (2.5 M sodium acetate) was added to each well to stop the reactions, and the fluorescence intensity was recorded using a Varioskan flash spectrophotometer plate reader at excitation and emission values of 545 nm and 590 nm, respectively. The IC_50_ and standard deviation values of both the test compounds and reference standard were calculated using the Graph Pad Prism.

### 2.6. Antioxidant Activity of ***2a**–**d*** and ***3a**–**d***

#### 2.6.1. Determination of the Reducing Activity of the Stable, Radical DPPH by **2a**–**d** and **3a**–**d**

The antioxidant activities of the test compounds against ascorbic acid (Sigma Aldrich, Saint Louis, MO, USA) as a positive control were evaluated using a 2,2-diphenyl-1-picrylhydrazyl (DPPH) radical scavenging assay developed by Zhu et al., as described in our previous study [26]. Duplicate solutions (20 μL) of the test compounds and ascorbic acid in DMSO (final concentrations: 5, 10, 25, 50, and 100 μM) were added into each designated well of a 96-well plate. A solution of DPPH (0.20 mM) (20 μL) in methanol was added to each well, and the 96-well plate was wrapped with aluminium foil and incubated in the dark for 45 min. Five absorbance readings were recorded at 512 nm using a Varioskan flash microplate spectrophotometer reader. The average values obtained from the absorbance readings were used to determine the IC_50_ and standard deviation values.

#### 2.6.2. Nitric Oxide Radical Scavenging Activity of Compounds **2a**–**d** and **3a**–**d**

Nitric oxide was generated from sodium nitroprusside and measured by Griess’ reaction following the literature method [27]. The experiment was done in triplicate with ascorbic acid (vitamin C) used as a positive control for the assay. In total, 50 μL of each of the test compounds and the positive control at various concentrations (0.1, 0.5, 1.0, 5.0, and 10.0 μM in methanol) were added to each designated well of the 96 well plate. A total of 50 μL of sodium nitroprusside (10 mM) prepared in saline phosphate buffer (pH = 7.4) was then added to each well and the reaction mixture incubated at room temperature for 3 h. Afterward, 300 μL of the Griess reagent (a mixture of 0.2% *N*-(1-naphthyl)ethylenediamine dihydrochloride and 2% sulfanilamide in 5%) was added to each well and absorbance was read at 546 nm using a Varioskan flash microplate spectrophotometer reader.

### 2.7. Enzyme Kinetic Studies of ***2a*** and ***3b*** against AChE, BChE, and β-Secretase

#### 2.7.1. Enzyme Kinetic Studies of **2a** and **3b** against AChE and BChE

The AChE and BChE enzyme kinetic studies were performed in triplicate in a 96-well plate following a procedure described in our previous study [26]. Varying concentrations of **2a** or **3c** (0, 2.5, 3.5, and 5 μM) and AChI or BChI (0.1, 0.5, 2.5, and 5 mM) were used for the kinetic assays. The absorbances were measured after every 1 s for 10 s at a wavelength of 412 nm using a microplate spectrophotometer reader. The Lineweaver–Burk plots (plot of the inverse of velocity (1/v) against the inverse of the substrate concentration (1/[S])) were constructed. Dixon plots (plot of 1/v against concentration of inhibitor at each concentration of substrate) were used to determine the inhibitor constant, K_i_.

#### 2.7.2. Enzyme Kinetic Studies of **2a** and **3b** on β-Secretase

Compounds **2a** and **3b** were subjected to kinetic studies on β-secretase using substrate concentrations 150, 300, and 450 nM at increasing inhibitor concentrations (0, 4, 8, and 16 μM) following a method reported before [26]. The fluorescence readings were recorded after every 1 s for 10 s at excitation and emission values of 545 nm and 590 nm, respectively. The Lineweaver–Burk plots (plots of the inverse of velocity against the inverse of the substrate concentration) were constructed to assess changes in enzymatic parameters. The Dixon plots were also prepared to determine the inhibitor constants, K_i_.

### 2.8. Metal Binding Studies of ***2a*** and ***3b***

The metal chelating ability of compounds **2a** and **3b** toward Cu^2+^ and Zn^2+^ was evaluated by ultraviolet–visible (UV–Vis) spectrometry. A solution of the test compound in methanol (30 μM, final concentration) alone or in the presence of CuCl_2_ or ZnCl_2_ (30 μM, final concentration) was stirred at RT for 30 min. The absorption spectra were recorded at room temperature on a PerkinElmer UV/Vis spectrometer (Perkin Elmer, Inc., Midrand, Johannesburg, South Africa) at wavelengths ranging from 200 to 1200 nm.

### 2.9. Molecular Modelling Studies

#### 2.9.1. Molecular Docking of **2a**, **2c**, and **3b**

The PDB structure codes 4EY7, 1P0I, and 1M4H representing AChE, BChE, and β-Secretase were prepared using the prepared protein protocol prior to docking using Discovery Studio software version v20.1.0.19295 (Accelrys, San Diego, CA, USA). The binding site x, y, and z coordinates used for docking were 8.97, −58.9, and −25.7 with a radius of 11 for 4EY7; 136, 116, 38.4 with radius of 12 for 1P0I and 20.6, 33.7, 23 with a radius of 12.5 for 1M4H. Compounds **2a**, **2c**, and **3b** were drawn and prepared using the prepared ligand protocol with default settings. Donepezil and quercetin were also drawn and prepared in the same way as the compounds and docked as reference standards. Docking was performed using the CDOCKER module and the top poses with the optimal CDOCKER and CDOCKER interaction energy, with no unfavourable interactions selected.

#### 2.9.2. Predication of Physicochemical Parameters for **2a** and **3b**

The compounds **2a** and **3b** were drawn on the interactive online platform of the Molinspration property calculator (https://www.molinspiration.com/cgi-bin/properties accessed on 13 November 2020) and properties determined. The Blood Brain Barrier (BBB) calculation was performed using the web service SwissADME (http://www.swissadme.ch/ accessed on 13 November 2020). Compounds SMILES files were used on this platform.

### 2.10. Evaluation of Cytotoxicity (MTT assay) of ***2a*** and ***3b*** on the Vero and A549 Cells

The cytotoxicity activity of the compounds was evaluated using the 3-(4,5-dimethylthiazol-2-yl)-2,5-diphenyltetrazolium bromide (MTT) assay following a modification of a method by Mosmann [28]. Normal monkey kidney cells (Vero cells) and adeno-carcinomic human epithelial cells (A549 cells) were maintained in Dulbecco’s modified Eagle’s medium (DMEM, Gibco) supplemented with 10% fetal bovine serum (FBS) and 1% penicillin/streptomycin in culture flasks and incubated at 37 °C in 5% CO_2_. When cells reached 85% confluency, they were detached using 2% trypsin and cell count was performed using a handheld automated cell counter (Scapter 3.0™, Merck, Burlington, MA, USA). Cells were seeded at 1 × 10^5^ cells/well and incubated overnight to allow cell attachment. After 24 h, treatments were administered with different concentrations (0, 2.5, 5, 10, 25, 50, and 100 μM) of the compounds and doxorubicin (positive control). After 24 h of incubation, 20 μL of the MTT solution (5 mg/mL) was added in all the wells, which was followed by 4 h of incubation. DMSO (100 μL) was then added to dissolve the formazan crystals and the absorbance was measured at 517 nm using a microplate reader (VarioSkan Flash, Thermo Fisher Scientific, Vantaa, Finland).

### 2.11. Measurement of Reactive Oxygen Species (ROS) in Cells

Mitochondrial ROS levels in Vero and A549 cell lines was measured using the 2′,7′-dichlorofluorescin diacetate (H_2_DCF-DA) (Sigma-Aldrich, St. Louis, MO, USA, Merck, Darmstadt, Germany), fluorescent probe [29]. Cells (1 × 10^5^) were seeded in 96 well plates and incubated overnight at 37 °C in 5% CO_2_. A minimum non-toxic concentration (10 μM) of compounds (**2a** and **3b**) was added separately as treatment and further incubated for an hour, after which LPS (1 μg/mL) was added to induce the production of ROS. After 24 h of incubation, the media was aspirated, cells were rinsed with PBS, and followed by the addition of H_2_DCF-DA (10 μM) for 30 min in the dark. The absorbance of the fluorescence was measured using a microplate reader (VarioSkan Flash, Thermo Fisher Scientific, Finland) at 485 and 535 nm values of excitation and emission, respectively. Data was processed and analysed using ANOVA, which was followed by Duncan’s multiple comparison test.

## 3. Results and Discussion

### 3.1. Chemical Synthesis

**Scheme 1:**
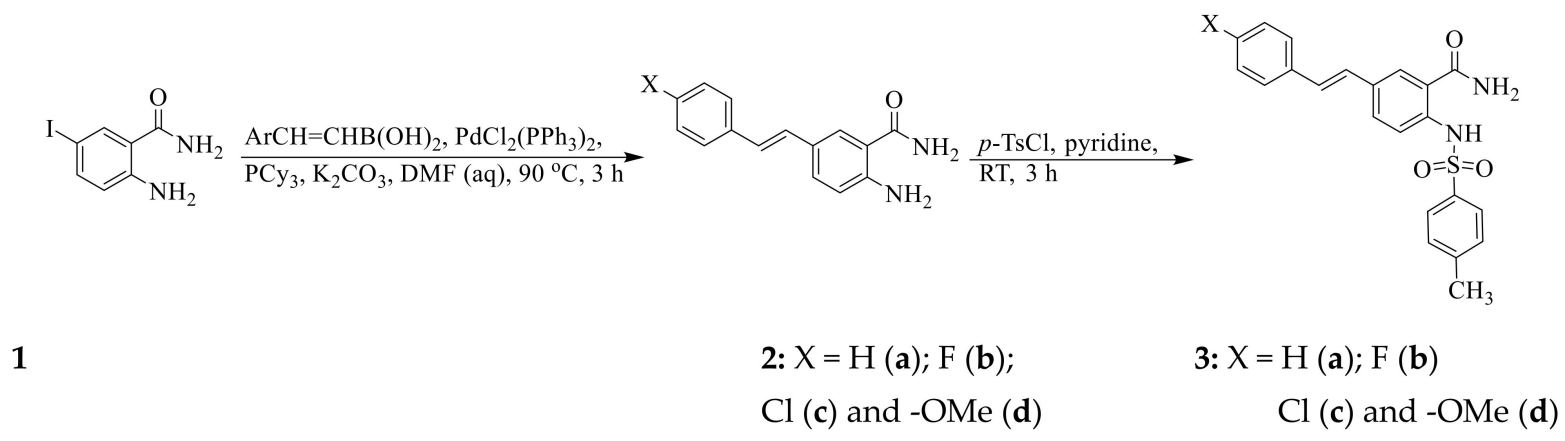
Synthesis of 5-styrylbenzamides **2a**–**d** and their sulfonamido derivatives **3a**–**d**.

The test compounds **2a**–**d** and **3a**–**d** were prepared as represented in Scheme 1 below. Initial Suzuki-Miyaura cross-coupling of 5-iodoanthranilamide (2-amino-5-iodobenzamide) (**1**) with styrylboronic acids afforded the corresponding 5-styrylbenzamides **2a**–**d**. Treatment of the latter with *p*-toluenesulfonyl chloride in pyridine at room temperature afforded the corresponding 5-styryl-2-(*p*-tolylsulfonamido)benzamide derivatives **3a**–**d**. The structures of the prepared compounds were characterised using a combination of ^1^H-NMR, ^13^C-NMR, IR, and mass spectrometric techniques. Copies of their NMR spectra are included as Figure S1 in the Supplementary Materials. The *E*-stereochemistry of the styryl arm of compounds **2** and **3** was confirmed by the presence of a set of doublets in the aromatic region of their ^1^H-NMR spectra with coupling constant (*J*_trans_) values of about 16.0 or 16.5 Hz. The amide hydrogen atoms of compounds **2** resonated as two distinct broad singlets of reduced intensity and different chemical shift values around 7.12–7.13 ppm and 7.82–7.90 pm similar to the literature observation for the corresponding anthranilamide precursors [30]. The observed chemical non-equivalence of these hydrogen atoms is attributed to the hindered rotation of the C(O)–NH_2_ single bond in terms of the NMR time scale. The hydrogen atoms of -C(O)NH_2_ of derivatives **3** are also non-equivalent with different chemical shift values. A singlet for NH of the 4-methylphenylsulfonamido group of derivatives **3** resonates significantly downfield due to the de-shielding effect of the S=O bonds. The downfield shift of this signal of the *N*^2^-sulfonamide derivatives is also consistent with the presence of an intramolecular hydrogen bond between this hydrogen and the carbonyl oxygen, as previously observed for the *ortho*-(4-tolylsulfonamido)benzamides [31]. Conformational restriction of small drug molecules due to intramolecular hydrogen bonding has been found to increase their lipophilicity, passive membrane permeability, and, therefore, brain penetration, as well as pharmacological activity due to favourable alignment with the protein pocket, which results in increased ligand-receptor interactions [32].

### 3.2. Biology

The 5-styrylbenzamides **2a**–**d** and their 5-styryl-2-(*p*-tolylsulfonylmido)benzamide derivatives **3a**–**d** were evaluated for a potential to inhibit cholinesterase enzymes (AChE and BChE) and β-secretase as well as for free radical scavenging activities using donepezil, quercetin, and ascorbic acid as reference standards, respectively (Table 1). A significant inhibitory effect against AChE activity was observed for the 5-styrylbenzamides **2a**–**d** with IC_50_ values 2.3 μM (**2a**), 5.4 μM (**2b**), 8.6 (**2c**), and 7.8 μM (**2d**) though weaker than that of selective, uncompetitive, and reversible acetylcholinesterase inhibitor, donepezil (IC_50_ = 1.24 ± 0.18 μM). The observed activity is likely due to the presence of amino and amide groups with an increased propensity to form hydrogen bonding interactions. The styryl and 4-methoxystyryl substituted *N*^2^-tosyl derivatives **3a** and **3d** exhibited moderate activity against AChE compared to the corresponding substrates with IC_50_ values of 9.5 μM and 11.3 μM, respectively. A combination of the *N*^2^-tosyl and 4-halogenostyryl group, on the other hand, resulted in a significant inhibitory effect against AChE activity for derivatives **3b** and **3c** with IC_50_ values of 4.3 μM and 7.8 μM, respectively. It has been observed that the level of AChE in the brain of AD patients decreases extensively as the disease progresses, while BChE activity is maintained at the normal level or increases [33]. Specific BChE inhibitors were able to restore ACh levels in mice and improve the cognitive impairment caused by the amyloid-β peptide [34]. Moreover, AChE knockout mice models indicated that BChE can potentially substitute for AChE, maintaining normal cholinergic pathways in AChE nullizygous animals [18]. Although selective inhibition of BChE activity could be advantageous for the treatment of advanced AD, it can also lead to adverse peripheral side effects. Tacrine, which is the first FDA approved drug for the treatment of AD, has more activity toward BChE than AChE and is hepatotoxic in nature [35]. An improved AD therapy could benefit from mixed inhibition of AChE/BChE enzymes. Evaluation of compounds **2a**–**d** against BChE activity revealed **2a** as the most active within this series compared to donepezil (2.98 ± 0.18 μM) with an IC_50_ value of 4.7 ± 0.32 μM. Compounds **2b**–**d**, which exhibited a significant inhibitory effect against AChE, were found to be less active against BChE. The *N*^2^-tosylated analogues **3a**–**d**, on the other hand, were found to exhibit moderate to significant activity against BChE with IC_50_ values ranging from 6.9 to 13.3 μM. The styryl and 4-methoxystyryl substituted *N*^2^-tosyl derivatives **3a** and **3d** exhibited reduced and moderate activity against this enzyme compared to their substrates. A combination of the *N*^2^-tosyl and 4-halogenostyryl group, on the other hand, resulted in a significant inhibitory effect for derivatives **3b** and **3c** against BChE compared to the corresponding precursors with IC_50_ values of 4.3 μM and 7.8 μM, respectively. The strong electron-withdrawing effect of the halogen atom could help the drug molecules in forming noncovalent bonding interactions with the protein targets, and, thus, enhance biological activity [36]. Compounds **2a**, **3b**, and **3c** with a dual inhibitory effect against AChE and BChE have the potential to block both the catalytic and peripheral anionic sites of AChE, and the catalytic activity of BChE. Such interactions will likely ameliorate AD symptoms with minimal or no side effects [32].

BChE was found to be enriched within Aβ plaques in the brains of AD patients while BChE-knockout reduces Aβ fibrils in the brain of an AD mouse model [37]. The test compounds are analogues of mefenamic acid (**D**) that has previously been found to improve learning and memory impairment in an Aβ-infused Alzheimer’s disease rat model [16]. Blocking the production of Aβ plaques through the inhibition of β-secretase 1 is an alternative therapeutic approach for AD. β-secretase inhibitors have been shown to lower Aβ levels in the brain of mice [38]. All of the test compounds were further evaluated for an inhibitory effect against β-secretase using quercetin (3,3′,4′,5,7-pentahydroxyflavanone) as a reference standard. This flavonoid derivative exhibits a significant inhibitory effect against β-secretase and results in reduced levels of Aβ in neurons [39]. A β-Secretase inhibitory assay revealed derivative **2c** as the most active (IC_50_ = 6.7 μM) among the test compounds, more so than that of quercetin (IC_50_ = 10.4 μM). A moderate-to-significant inhibitory effect against β-secretase was observed for the tosylamido derivatives **3a**–**d** with IC_50_ values ranging from 10.1 to 19.3 μM. Transformation of the aniline nitrogen into sulfonamide has resulted in reduced anti-cholinesterase inhibitory activity for **3a**, though it has shown potent inhibition of BACE-1 than **2a**. The inhibitory effect of the 5-(4-fluorostyryl) substituted derivative **3b** against β-secretase (IC_50_ = 10.1 μM) is comparable to that of quercetin.

The test compounds were also evaluated for antioxidant activity in vitro through the DPPH and NO radical scavenging assays. DPPH radical scavenging assay confirmed the potential dual anticholinesterase and β-secretase inhibitor **2a** to exhibit significant free radical scavenging properties compared to ascorbic acid (IC_50_ = 4.2 μM) with an IC_50_ value of 5.8 μM. This compound also exhibited moderate NO scavenging properties against ascorbic acid (IC_50_ = 6.2 μM) with an IC_50_ value of 10.3 μM. The 5-(4-fluorostyryl) derivative **3b** with dual cholinesterase and β-secretase inhibitory properties, on the other hand, was found to exhibit reduced free radical scavenging activity in the DPPH (IC_50_ = 25.6 μM) and NO (IC_50_ = 15.8 μM) scavenging assays. The presence of a bulky methoxy group at the para position of the styryl arm of **2d** and **3d** appear to be less favourable for biological activity of these 5-styrylanthranilamide derivatives against the enzymes linked to AD.

Small metal chelators are promising candidates to target Aβ species in the inhibition of AChE or BChE to become potential AD therapeutics. We postulated that the presence of NH positioned ortho to the amide moiety could introduce a metal-chelating property that would be useful in the design of multi-target-directed ligands (MTDLs) for the treatment of AD. The most active compound against AChE and/or BChE, and β-secretase with increased free radical scavenging properties, namely, **2a** was selected for investigation of the metal chelating capacity in the presence of Zn^2+^ and Cu^2+^ in methanol.

### 3.3. Metal Chelation Study of Compound ***2a***

**Figure 2:**
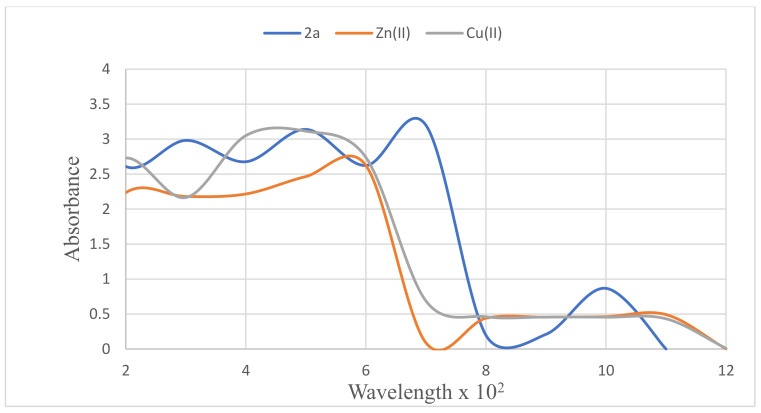
Ultraviolet (UV) spectra of compounds **2a** alone (30 μM, final concentration) or in the presence of various salts CuCl_2_ or ZnCl_2_ in methanol (final concentrations of 30 μM) at room temperature.

The ability of compound **2a** to chelate Cu^2+^ or Zn^2+^ was assessed using a UV–vis spectrophotometric assay with a wavelength in the region of 200–1200 nm. The absorption spectrum of **2a** (Figure 2) acquired in methanol at room temperature is characterized by four discernible absorption bands, around λ 300, 500, 690, and 1000 nm, respectively. These bands are reduced to two distinct bands of different intensities in the presence of either CuCl_2_ or ZnCl_2_. Addition of CuCl_2_ to a solution of **2a** resulted in a significantly broad absorption maximum around 490 nm, while the maximum at 1000 nm shifted to around 1104 nm, indicating the formation of a ligand-Cu^2+^ complex. A bathochromic shift of the band found around 500 nm in the spectrum of **2a** to around 580 nm in the presence of ZnCl_2_ occurred with no broadening. 2-Aminobenzamide is known to bind the Cu(II) ion through the amino-N and amido-O atoms [40]. These preliminary results suggest **2a** to be selective for Cu (II) ion and to have relatively poor chelating ability for Zn^2+^.

Compound **2a** with inhibitory activity against cholinesterases and β-secretase as well as antioxidant and metal chelating potential was selected for kinetic studies against these enzymes.

### 3.4. Kinetic Studies of ***2a*** on AChE, BChE, and β-Secretase

**Figure 3:**
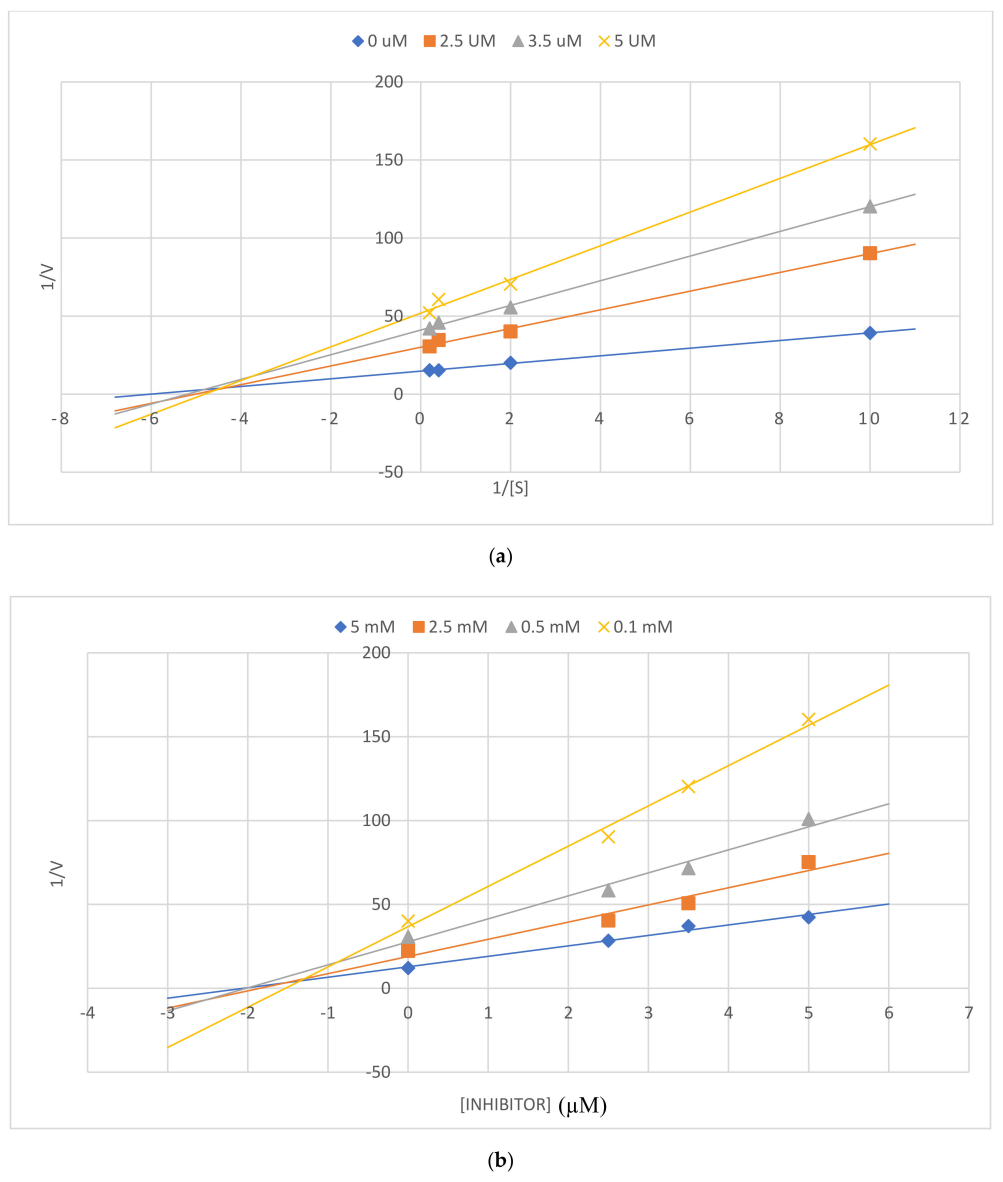
Lineweaver-Burk plot (**a**) and Dixon plot (**b**) of **2a** against AChE.

**Figure 4:**
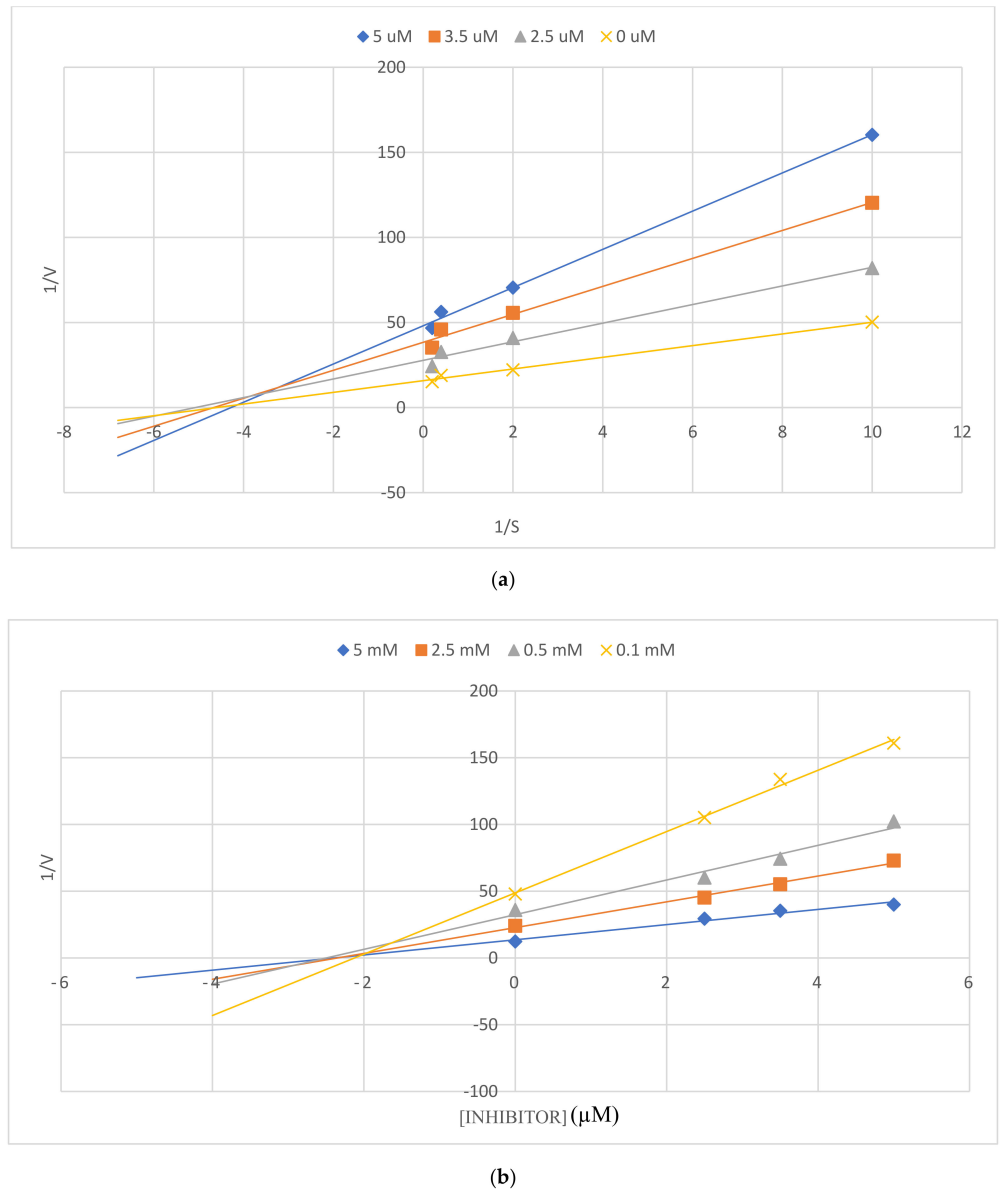
Lineweaver-Burk plot (**a**) and Dixon plot (**b**) of **2a** against BChE.

The cholinesterase (AChE and BChE) enzyme kinetics on **2a** was studied at increasing inhibitor concentrations of 0, 2.5, 3.5, and 5 μM and substrate concentrations of 0.1, 0.5, 2.5, and 5.0 mM. The Lineweaver–Burk plot of **2a** against AChE (Figure 3a) shows a decrease in the V_max_ values from 0.068–0.019 μM/min and relatively unchanged K_m_ value of 0.0195 ± 0.02 μM, indicating a non-competitive mode of inhibition. This is confirmed by the Dixon plot (Figure 3b), which displays sets of straight lines intersecting on the x-axis with a Ki value of 1.2 μM. The Lineweaver–Burk plot of compound **2a** for BChE (Figure 4a) shows a decrease in V_max_ values (0.038–0.016 μM/min) with a relatively unchanged K_m_ (0.20 ± 0.11) value. Its Dixon plot (Figure 4b) shows several straight lines that intersect just above the x-axis with a Ki value of 2.0 μM. This compound, therefore, exhibits a mixed mode of inhibition against BChE.

### 3.5. Kinetic Study of ***2a*** on β-Secretase

**Figure 5:**
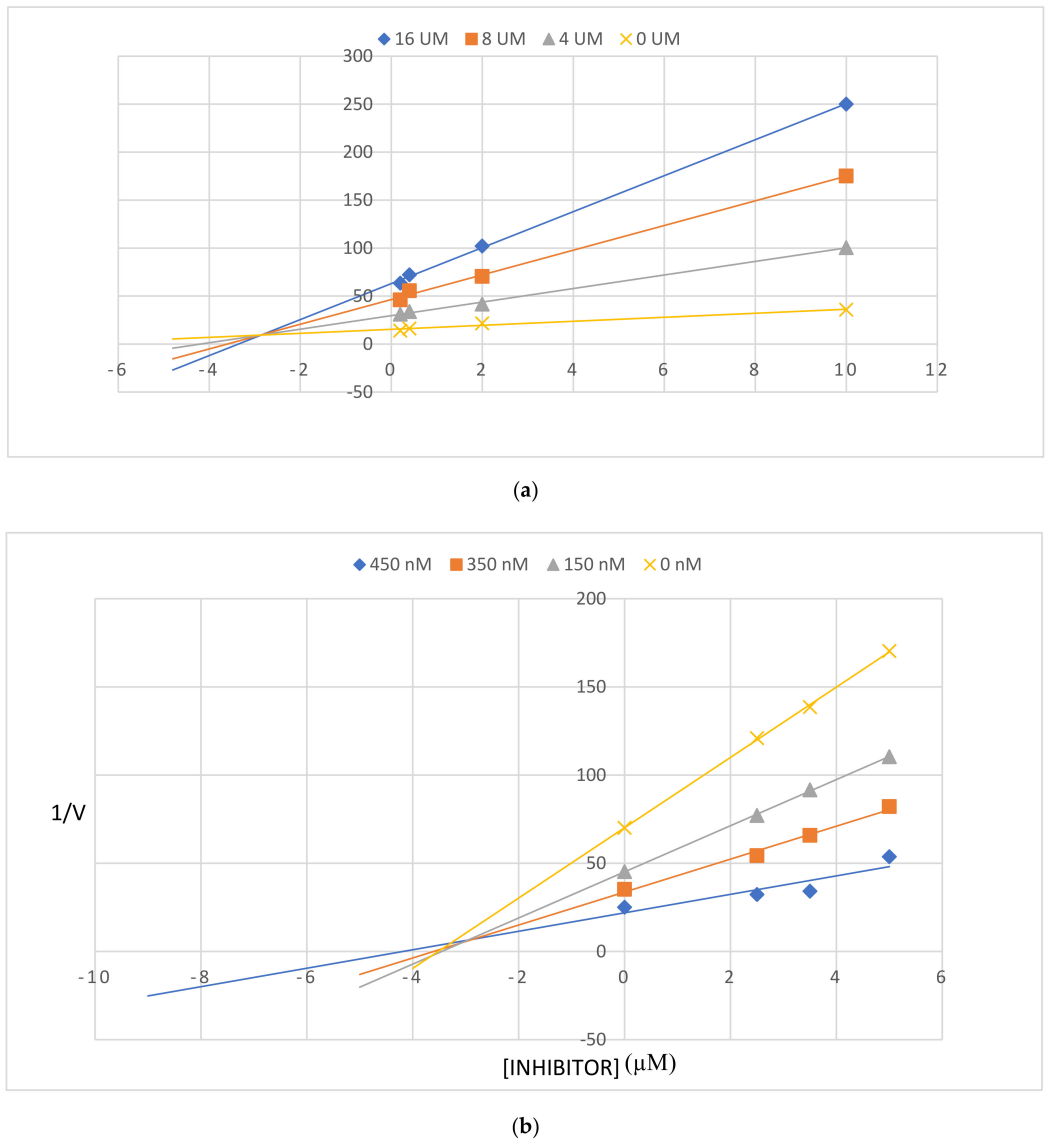
Lineweaver-Burk plot (**a**) and Dixon plot (**b**) of **2a** against β-secretase.

The enzyme kinetics of **2a** against β-secretase was evaluated at increasing inhibitor concentrations of 0, 4, 8, and 16 μM, and substrate concentrations of 0, 150, 300, and 450 nM. The Lineweaver-Burk plot of this compound (Figure 5a) is characterised by decreasing V_max_ (0.065–0.02 μM/min) and an unchanged K_m_ value of 0.03 + 0.01. Its Dixon plot (Figure 5b), on the other hand, shows a set of straight lines that intersect just above the x-axis with a Ki value of 3.44 ± 0.3 μM. The observed trends are consistent with a mixed mode of inhibition of this compound against β-secretase.

In an attempt to rationalize the structure activity relationship and to figure out the plausible protein-ligand interactions at a molecular level, we performed molecular docking studies of compounds **2a** and **3b** into the binding sites of AChE (PDB code 4EY7) and BChE (1P0I). Compounds **2a**, **2c**, and **3b** were also docked into the active site of β-secretase (1M4H).

### 3.6. Computational Studies

#### 3.6.1. Docking of **2a** and **3b** into Cholinesterase Enzyme (AChE and BChE) Binding Sites

**Figure 6:**
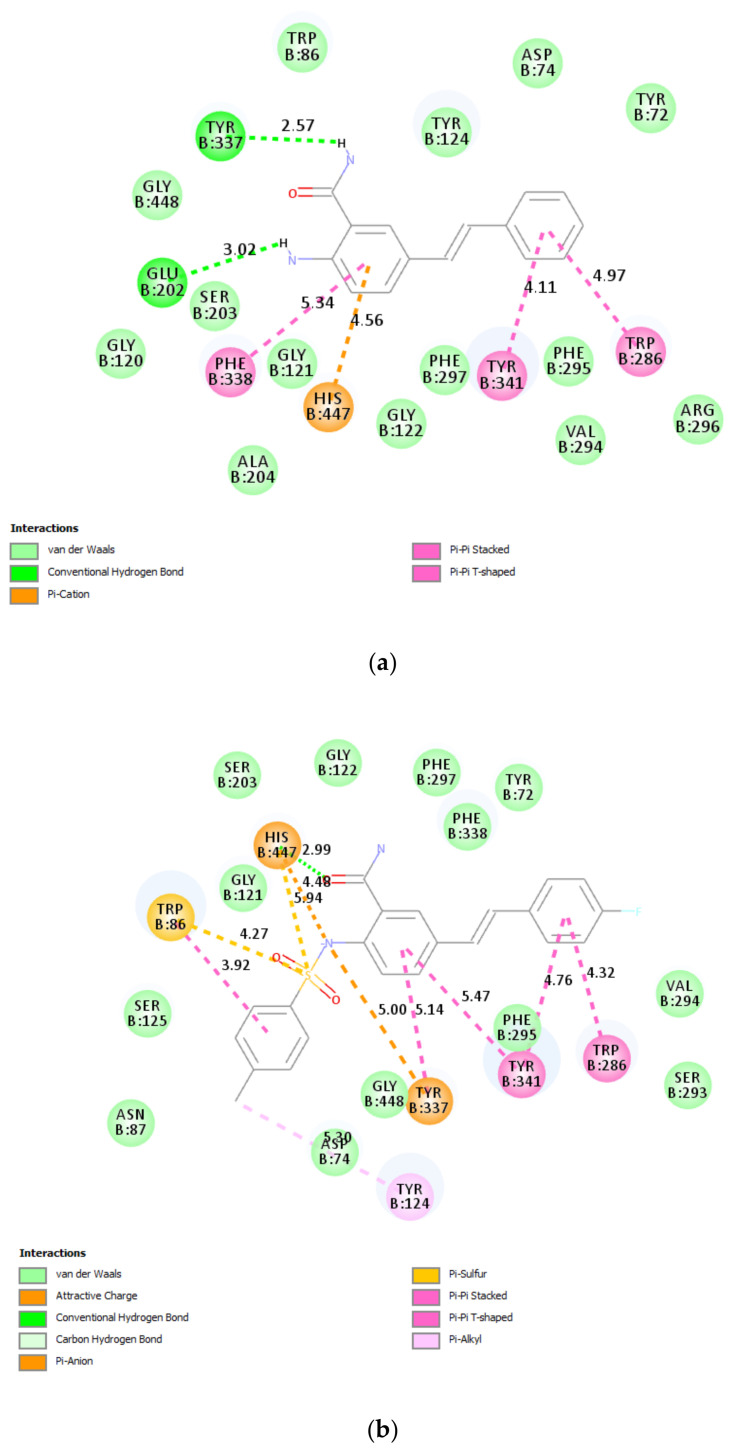
The 2-dimensional (2D) plots of docking results for **2a** (**a**) and **3b** (**b**) into AChE including bonding interaction distances and colour codes for various interactions.

Both AChE and BChE possess two binding sites, namely, the catalytic site (CAS) and peripheral anionic site (PAS) with high binding affinity towards substrates and inhibitors. The main difference between the two enzymes is associated with the acyl binding site, which is significantly bigger for BChE compared to that of AChE. Donepezil was docked into the active site of the AChE crystal (PDB code: 4EY7) and the top scoring docked pose was applied as a starting point for molecular dockings (refer to Figure S2 in Supplementary Materials). Donepezil interacts with both CAS and PAS tryptophans of AChE via ring-stacking interactions, which are predicted between its fused benzo ring and the residues Trp286 and Tyr341, as well as between the phenyl substituent and Trp86. These predicted interactions agree with those seen in the structure of ACHE with donepezil co-crystalised (4EY7). Electrostatic (π-cation and charge transfer) interactions exist between its nitrogen atom and the protein residues Asp74, Trp86, and Tyr337. The carbonyl oxygen of donepezil is involved in a hydrogen bonding interaction with Phe295 in the docked structure as well as the crystal structure. Compounds **2a** and **3b** were docked individually into the active site of AChE using the same parameters and site as for the docking of donepezil. The docking pose of **2a** (Figure 6a) shows the presence of a π-π interaction between the anthranilamide ring and the protein residue Phe338 in the choline-binding site, and also between the phenyl ring and Tyr341 of PAS. The phenyl ring of this compound is also involved in a T-shaped π-π stacking interaction with Trp286 of PAS. The electrostatic (π-cation) interaction is predicted between the anthranilamide ring and His447 of CAS. The amino group of this compound is envisioned to be involved in a hydrogen bonding interaction with Glu202, and a similar interaction is also predicted between the amide hydrogen with Tyr337 of the choline binding site. The predicted strong interactions of **2a** with protein residues in PAS of AChE are consistent with the inhibitory effect and the observed non-competitive kinetic type of inhibition for this compound, allowing the substrate to bind in the large active site gorge, but decreasing the enzymatic activity by its position. Such compounds have the potential to prevent the pro-aggregating activity of AChE toward Aβ [41]. The styryl moiety of the second most active derivative **3b** is predicted to be involved in aromatic-aromatic (π-π stacked and π-π T-shaped) interactions with Trp286, Tyr337, and Tyr341 (Figure 6b) of AChE. The nitrogen atom of the sulfonamide group is involved in an attractive charge interaction with Tyr337 in the choline binding site and also π-anion bonding interaction with and His447 of CAS. The latter residue is also involved in a hydrogen bonding interaction with the amide oxygen and π-sulfur bonding interaction with the sulfur atom. The sulfur atom is involved in π-sulfur interaction with His447 and Trp86, and the latter residue also interacts with the *p*-tolyl ring through a π-π stacking interaction. The methyl group of the *p*-tosyl moiety is involved in a π-alkyl interaction with Tyr124.

**Figure 7:**
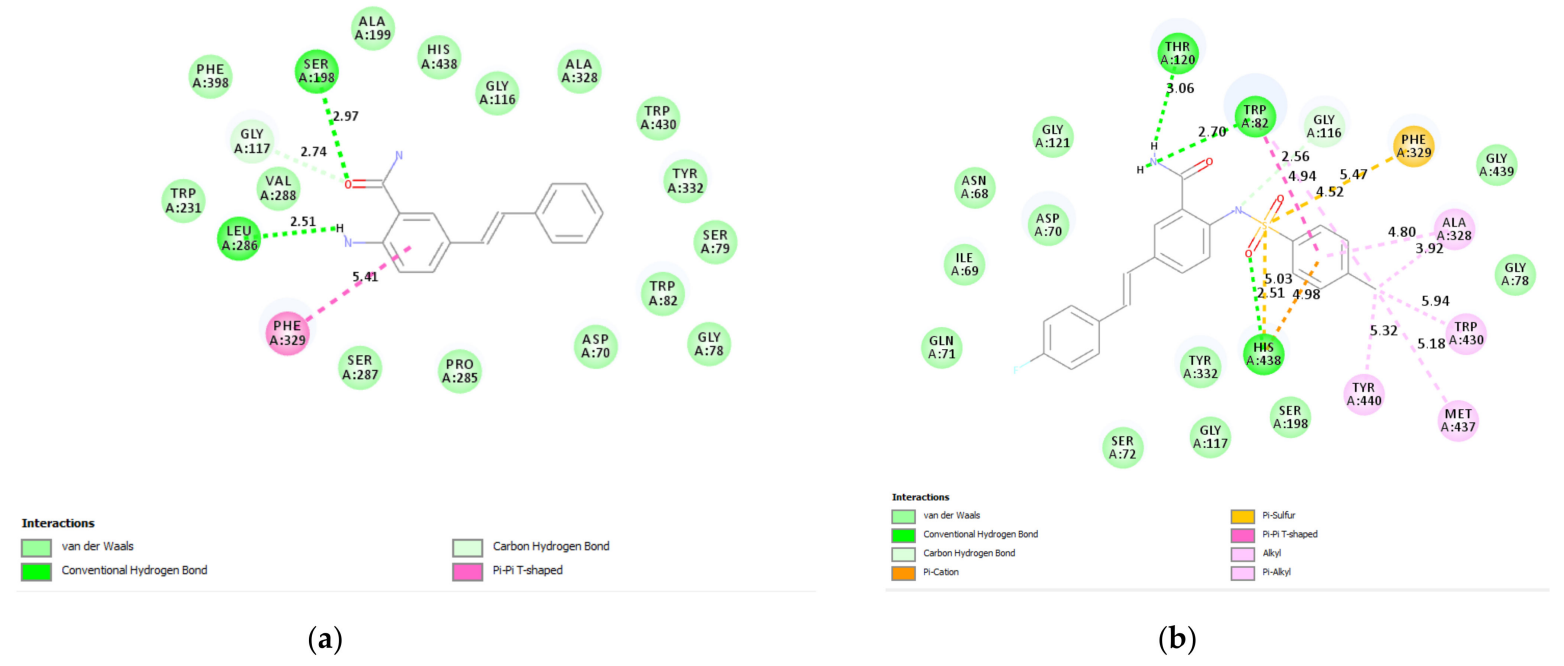
The 2-dimensional (2D) plots of docking results for **2a** (**a**) and **3b** (**b**) into BChE including bonding interaction distances and colour codes for various interactions.

No hydrogen bonding interaction is predicted between donepezil and the protein residue of BChE (see Figure S2 in Supplementary Materials). The styryl wing of **2a** is predicted to not be involved in any interaction with the protein residues in the active site of BChE (Figure 7a). However, there is an aromatic-aromatic (π-π T-shaped) interaction between its anthranil-amide ring and Phe329 in the acyl-binding pocket. The carbonyl oxygen of this compound is involved in a weak carbon hydrogen bonding interaction with Gly117, and also a hydrogen bonding interaction with Ser198 of the catalytic triad. The amino group of this compound is predicted to be involved in a hydrogen bonding interaction with Leu286. These interactions explain the observed mixed mode inhibitory effect of **2a** against BChE as this compound binds to the active site as well as other sites on the enzyme to affect its activity against this enzyme. The interactions of **2a** with several residues in PAS of AChE, and Ser198 of BChE, make it a potential dual anticholinesterase inhibitor with capability to block the PAS of AChE and the catalytic activity of BChE. No interactions are predicted between the styryl framework of **3b** and any of the protein residues in BChE (Figure 7b). However, the hydrogen atoms of amide nitrogen are involved in hydrogen bonding interactions with Thr120 and Trp82. Incorporation of the *p*-tolylsulfonamide moiety resulted in several interactions. Alkyl and π-alkyl interactions exist between its methyl group and protein residues Ala328, Met437, Trp82, Trp430, and Tyr440. The ring of the tosyl group is involved in a π-alkyl interaction with Ala328 and π-π stacking as well as a T-shaped π-π interaction with Trp82. Interaction with Trp82 determines the selectivity toward BChE [42] and potential for dual-binding inhibition [43]. His438 of CAS is involved in a π-cation interaction with the tosyl ring and also π-sulfur interaction with the sulfur atom as well as a hydrogen bonding interaction with the oxygen atom of the sulfonamide moiety. There is an additional π-sulfur interaction between the sulfur atom and Phe329.

#### 3.6.2. β-Secretase **2a**, **2c**, and **3b**

**Figure 8:**
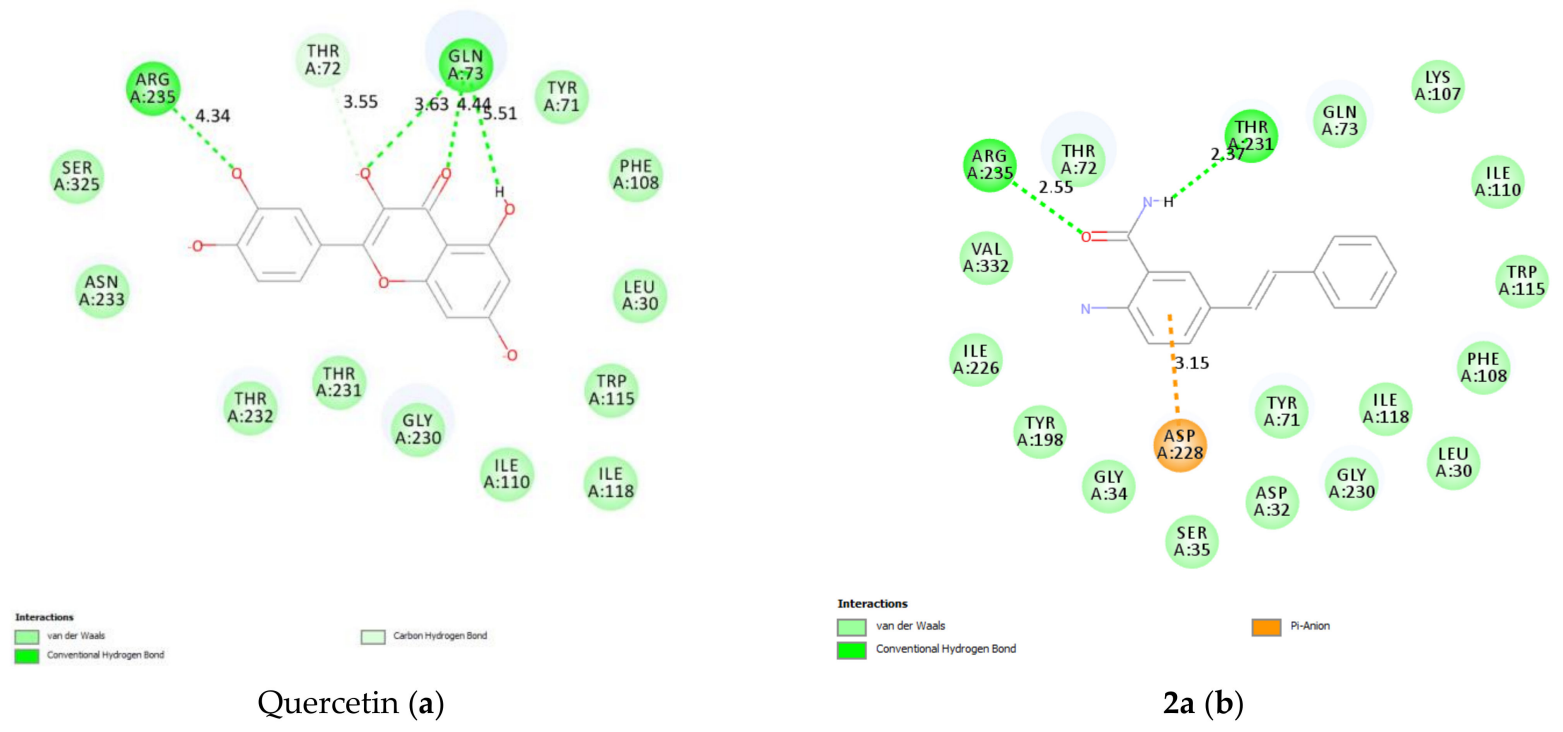

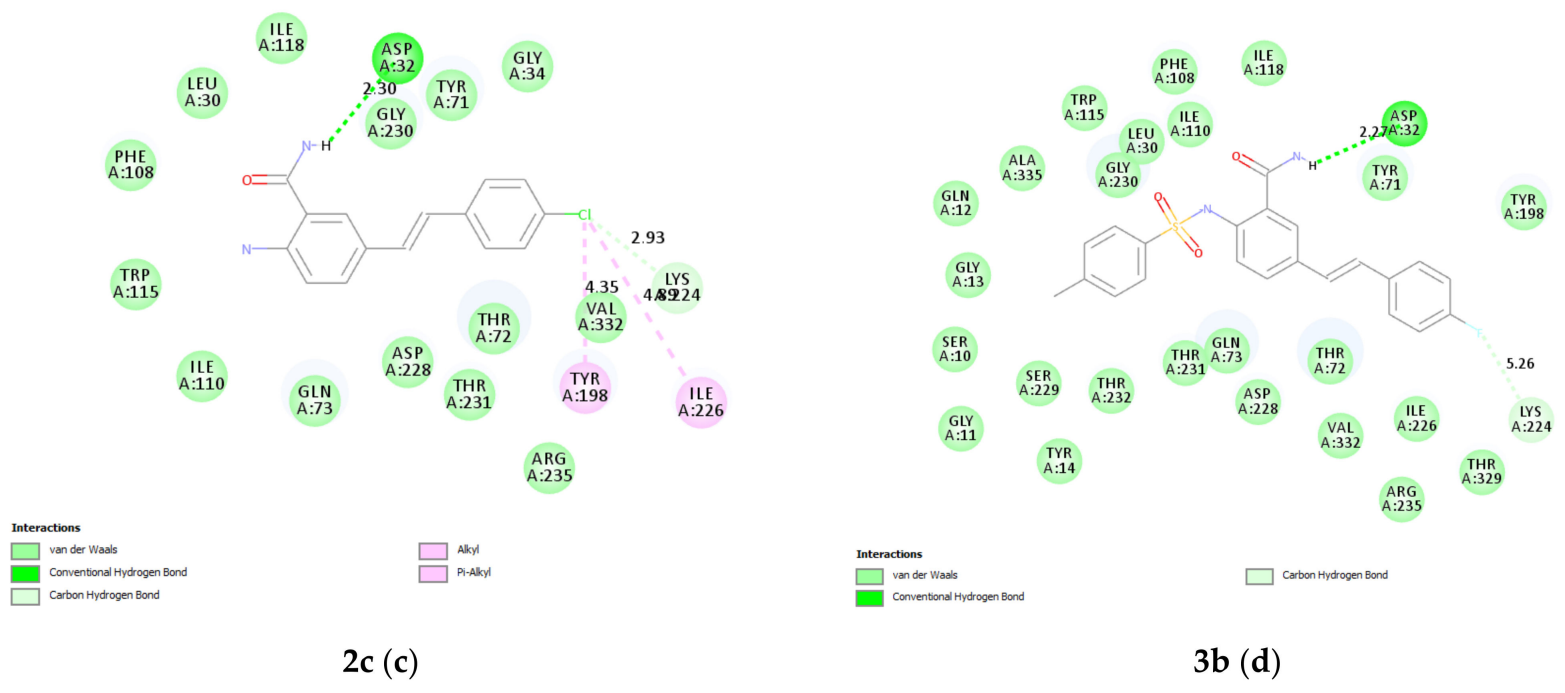
The 2-dimensional (2D) plots of docking results for quercetin (**a**), **2a** (**b**), **2c** (**c**), and **3b** (**d**) into β-secretase including bonding interaction distances and colour codes for various interactions.

The catalytic domain of β-secretase contains eight pockets with different amino acid residues [44], and these pockets enable different inhibitors to bind to different sites and a few sites at the same time [45]. The extended active site and inherent flexibility of β-secretase, on the other hand, make it a difficult target for inhibitors compared to the cholinesterase enzymes [46]. Inhibitors of β-secretase are required to be large enough to interact within the large active site of this enzyme, and be small enough to exhibit suitable drug-like properties [47]. Quercetin has been reported from in vitro and in silico studies to inhibit BACE-1 enzyme activity through the formation of hydrogen bonds [39]. This flavonoid derivative used as a reference standard in the assay was docked into the active site of β-secretase (PDB code: 1M4H), and the top scoring docked pose was applied as a starting point for molecular docking of the test compounds (Figure 8a). Quercetin makes 3 hydrogen bonds with Gln73 and one hydrogen bond with Arg235 as well as a carbon hydrogen bond Thr72. The benzene ring of benzamide scaffold of **2a** is predicted to be involved in a π-anion interaction with the catalytic aspartic acid residue Asp228 (Figure 8b). Two hydrogen bonding interactions are also predicted between the amide oxygen and hydrogen atoms of this compound with Arg235 and Thr231, respectively. The styryl arm of this compound seems not to be involved in an interaction with any of the protein residues of β-secretase, which may account for this compound’s slightly reduced inhibitory effect against this enzyme. This compound’s binding likely affects the conformational dynamics of the protein and the enzyme’s ability to catalyze the reaction as well as possibly blocking the substrate binding to the active site, which agrees with our kinetic data by showing a mixed type of inhibition. The chlorine atom on the styryl wing of **2c** is predicted to be involved in several hydrophobic interactions (van der Waals, alkyl, π-alkyl) with Tyr198 and Ile226 as well as a carbon hydrogen bonding interaction with Lys224 (Figure 8c). The benzamide scaffold extends into the hydrophobic S1 pocket but makes no interaction with any of the protein residues (Leu30, Tyr71, Phe108, and Trp115) in this pocket. However, this orientation facilitates a hydrogen bonding interaction of the amide hydrogen atom with the catalytic Asp32 presumably consistent with the observed increased inhibitory effect of this compound against β-secretase. The Asp32 and Asp228 residues in BACE-1 play a key role in catalyzing the hydrolysis of amide bonds of the amyloid precursor protein (APP), which represent the catalytic sites of BACE-1. A fluorine atom on the styryl wing of **3b** is involved in a carbon hydrogen bonding interaction with Lys224 (Figure 8d). Despite fitting into the large catalytic pocket of β-secretase, the 2-(*p*-tolylsulfonamido) group of **3b**, which is known to establish strong electrostatic and hydrogen bonding interactions with various biochemical targets [24], does not interact with any of the residues of this enzyme. Although no hydrophobic interactions are predicted between this compound and any of the residues in the binding sites of β-secretase, its amide hydrogen atom is the core functional group involved in a hydrogen bonding interaction with Asp32 of the catalytic dyad. This hydrogen bonding interaction and carbon hydrogen bonding interaction with the fluorostyryl arm likely account for a slightly higher inhibitory activity of this compound against this enzyme compared to **2a**. On the other hand, no interaction of quercetin with a catalytic dyad (Asp32 and/or Asp228) is predicted, which may explain its relatively weak BACE-1 inhibition activity compared to **2c** and **3b**.

### 3.7. Prediction of ADME Descriptors for ***2a*** and ***3b***

The drug’s success depends on pharmaco-physical properties such as oral absorption, blood-brain barrier penetration, toxicity, metabolism, aqueous solubility, logP, pKa, half-life, and plasma protein binding. Drug-likeness is a complex balance of various molecular properties, such as hydrophobicity, electronic distribution, hydrogen bonding characteristics, molecule size, and flexibility and presence of various pharmacophoric features [48]. The CNS active drugs generally have lower molecular weight (MW < 500), have moderate hydrophobicity (logP < 5), have fewer hydrogen bond donors and acceptors (HBD < 3 and HBA < 7), fewer rotatable bonds (RB < 8), and are less polar (polar surface area PSA < 70 Å^2^) than drugs that are not active in CNS [49]. The theoretically determined ADME parameters for compounds **2a** and **3b** revealed that these compounds fulfil all the four drug-likeliness characteristics (Table 2). The predicted absorption rate of nearly 85% also suggests possible oral administration for these compounds. Molecules with moderate polarity (PSA < 79 Å) and lipophilicity (logP from + 0.4 to + 6.0), on the other hand, have a high probability to cross the BBB by passive diffusion and access the CNS [50]. Compound **2a** has a high probability to cross the BBB by passive diffusion and access the CNS than **3b**.

**Figure 9:**
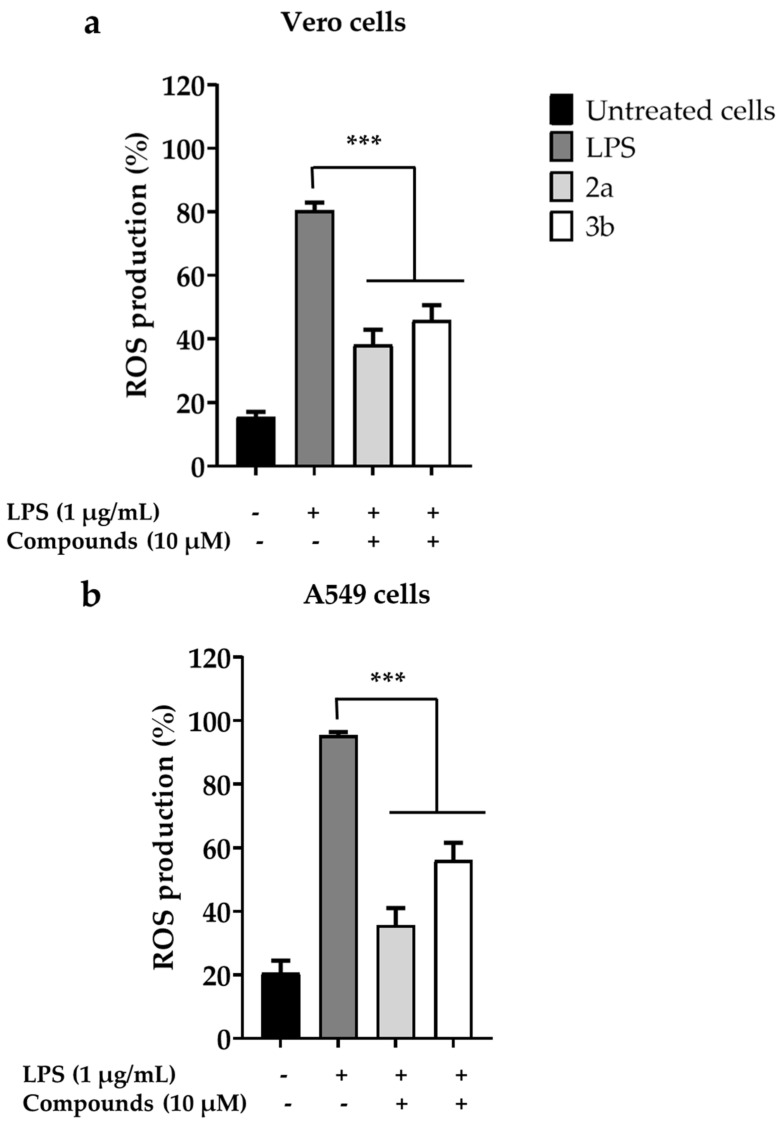
Effects of 2a and 3b on LPS-induced ROS production in Vero (**a**) and A549 cells (**b**). Data is expressed as mean ± SD (n = 3). Symbols * indicate a significant difference from untreated and treated cells. The *p*-value was calculated against LPS treatment, *** *p* < 0.01.

Considering that AD requires a stable long-term therapy, is well tolerated, and has low toxicity, most compounds **2a** and **3b** were evaluated for cytotoxicity against the Vero cells using the MTT assay to establish their safety profile. Both compounds did not affect the vaibility of these cells (Table 3). However, compounds **2a** and **3b** exhibited moderate and reduced cytotoxicity against the A549 cells compared to doxorubicin as a reference standard (IC_50_ 1.14 ± 0.23 μM) with IC_50_ values of 35.40 ± 0.13 μM and 55.00 ± 0.11 μM, respectively. Based on these cytotoxicity results, one concentration less than the IC_50_ (10 μM) that does not kill the cells was selected to evaluate the compounds’ suppression effects of LPS-induced ROS production. When LPS was added to the Vero and A549 cells, respectively, the amount of ROS produced increased dramatically (Figure 8). Compounds **2a** and **3b** in the presence of LPS supressed about 60% and 40% of ROS in the Vero and A549 cells, respectively (Figure 9a). Moderate ROS reduction was observed for **2a** (about 65%) while **3b** showed significant ROS reduction in the A549 cells (Figure 9b). These preliminary in vitro cell-based results are consistent with the good NO free radical scavenging activities of these compounds.

## 4. Conclusions

The metal chelating 2-aminobenzamides and potential anti-cholinesterase styryl moiety were combined to afford the 5-styrylbenzamides, and the latter transformed into corresponding 2-(4-fluorobenzamido)-5-styrylbenzamides and 2-(*p*-tolylsulfonamido)-5-styrylbenzamide derivatives. Among the test compounds, the 5-styrylbenzamide **2a** and **3b** displayed an inhibitory effect against cholinesterases (AChE and/or BChE) and β-secretase as well as free radical scavenging properties and metal chelating potential. The results of in vitro biological evaluation together with computational analyses present the 2-aminobenzamide moiety as a valuable chemical template, which deserves further optimization for an anti-AD drug discovery. The docking studies of the title compounds into the ChE and β-secretase active sites revealed an increased interaction of the benzamide moiety with several protein residues in the binding pockets of these enzymes. Molecular docking confirmed aromatic-aromatic (π-π stacked, π-π T-shaped) and hydrogen bonding interactions to be the key players responsible for anchoring the ligand in the active sites of the cholinesterase enzymes. The amide moiety is the core functional group involved in a hydrogen bonding interaction with protein residues in the active sites of the cholinesterase enzymes. A combination of a sulphonamide moiety and halogenostyryl arm on the anthranilamide scaffold has improved the activity of **3b** against all three targets, namely, AChE, BChE, and BACE-1. The compounds were found to exhibit moderate cytotoxicity against the A549 cell line and did not affect the viability of the Vero cells. Compounds **2a** and **3b** could be helpful in delaying or preventing AD, and also reduce the toxic effects of oxidative stress. These compounds exhibit strong antioxidant activity and will likely suppress many inflammatory effects mediated by oxidative stress with minimal cytotoxic effects on normal cells. For identifying a multi-targeted lead from this scaffold, the halogenated styryl arm appears to be important, and, therefore, future lead optimization should be targeted at modifying the position and number of halogen atoms on this wing to improve the activity against BACE-1 without compromising anti-cholinesterase activity. Further optimizations involving transformation of the anthranilamide scaffold into quinazolinone derivatives, on the other hand, will likely achieve novel, small, molecular BACE1 inhibitors with improved potencies and anticholinesterase activity. Cellular-based studies including bioavailability and cell permeability would help to clarify the mechanism of action of these compounds in the body and to establish their safety profile as potential multi-target agents against AD.

## Figures and Tables

**Table 1:** Inhibition of acetylcholinesterase (AChE), butyrylcholinesterase (BChE), and β-secretase, and antioxidant activities of **2a**–**d** and **3a**–**d**.

Compound	[IC_50_ (SD) μM]				
	AChE	BChE	β-Secretase	DPPH	NO
**2a**	2.3 ± 0.11	4.7 ± 0.32	15.8 ± 0.26	5.8 ± 0.32	10.3 ± 0.12
**2b**	5.4 ± 0.12	19.7 ± 0.25	13.9 ± 0.25	12.9 ± 0.22	15.8 ± 0.30
**2c**	8.6 ± 0.42	18.9 ± 0.24	6.7 ± 0.18	11.3 ± 0.11	9.3 ± 0.13
**2d**	7.8 ± 0.30	14.6 ± 0.47	17.2 ± 0.31	20.8 ± 0.24	11.2 ± 0.22
**3a**	9.5 ± 0.53	10.8 ± 0.40	16.5 ± 0.31	19.4 ± 0.40	13.1 ± 0.43
**3b**	4.3 ± 0.48	8.1 ± 0.52	10.1 ± 0.18	25.6 ± 0.30	7.6 ± 0.26
**3c**	7.8 ± 0.21	6.9 ± 0.10	15.0 ± 0.16	22.4 ± 0.51	8.6 ± 0.30
**3d**	11.3 ± 0.23	13.3 ± 0.26	19.3 ± 0.24	9.1 ± 0.26	12.3 ± 0.31
Donepezil	1.24 ± 0.15	2.98 ± 0.18	-	-	-
Quercetin	-	-	10.4 ± 0.32	-	-
Ascorbic acid	-	-	-	4.18 ± 0.13	6.23 ± 0.13

IC_50_ values (μM) were calculated from log dose inhibition curves and are expressed as means ± standard deviation (SD) of three independent experiments.

**Table 2:** Pharmacokinetics properties’ predictions of compounds **2a** and **3b**.

Property	2a	3b
miLogP	2.73	4.62
Topological polar surface area (Å)	69.12	89.26
Absorption (%)	85.15	78.21
Number of atom	18	29
Molecular weight	238.29	410.47
Molecular volume	224.43	349.88
Hydrogen bond acceptor	3	5
Hydrogen bond donor	4	3
Rotatable bonds	3	6
Lipinski’s violation	0	0
Blood-brain barrier (BBB)	Yes	No

miLogP: logarithm of partition coefficient of compound between *n*-octanol and water. ABS percentage = 109 (0.345 × Topological polar surface area) [51].

**Table 3:** The cytotoxicity of compounds **2a** and **3b** on Vero and A549 cell lines.

	IC_50_ (μM)	
Compound	Vero Cells	A549 Cells
**2a**	163.00 ± 0.55	35.40 ± 0.13
**3b**	191.40 ± 0.60	55.00 ± 0.11
**Doxorubicin**	0.66 ± 0.12	1.14 ± 0.23

## Data Availability

The data presented in this study are available on request from the corresponding author.

## Supplementary Materials

The following are available online at https://www.mdpi.com/article/10.3390/antiox10050647/s1, Figure S1: copies of the ^1^H- and ^13^C-NMR spectra of compounds **2**–**4** and Figure S2: the 2-dimensional (2D) plots of docking results for donepezil into AChE and BChE.
