# Supplementary material for: In Vitro Enzymatic and Kinetic Studies, and In Silico Drug-Receptor Interactions, and Drug-Like Profiling of the 5-Styrylbenzamide Derivatives as Potential Cholinesterase and β-Secretase Inhibitors with Antioxidant Properties
Source: Antioxidants (Basel). 2021 Apr 22;10(5):647. doi: 10.3390/antiox10050647 (PMC8145986; doi:10.3390/antiox10050647)
Supplement: Supplementary file 1 [file antioxidants-10-00647-s001.zip › antioxidants-1160956-supplementary.pdf]

Supplementary Information

In vitro enzymatic and kinetic studies, and *in silico* drug-receptor interactions and drug-like profiling of the 5-styrylbenzamide derivatives as potential cholinesterase and β-secretase inhibitors with antioxidant properties

Malose J. Mphahlele, [1,*] Emmanuel N. Agbo, [1] Garland K. More [2] and Samantha Gildenhuys [2]

Figure S1: ^{1}H- and ^{13}C-NMR of compounds **2a–d** and **3a–d**

Figure S2: The 2-dimensional (2D) plots of docking results for donepezil into AChE and BChE

Figure S1: ^{1}H- and ^{13}C-NMR of compounds **2** and **3**.

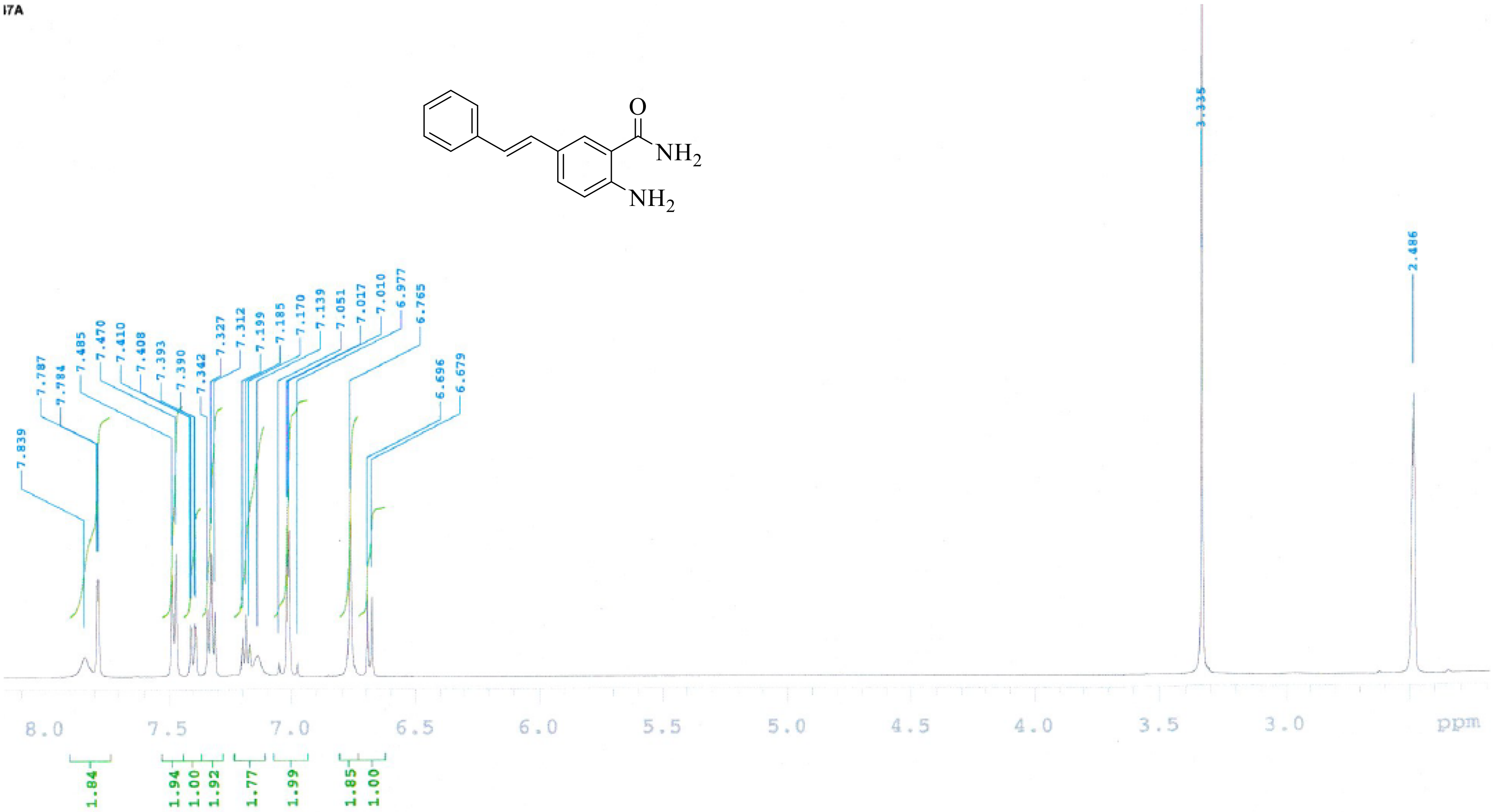

(a)

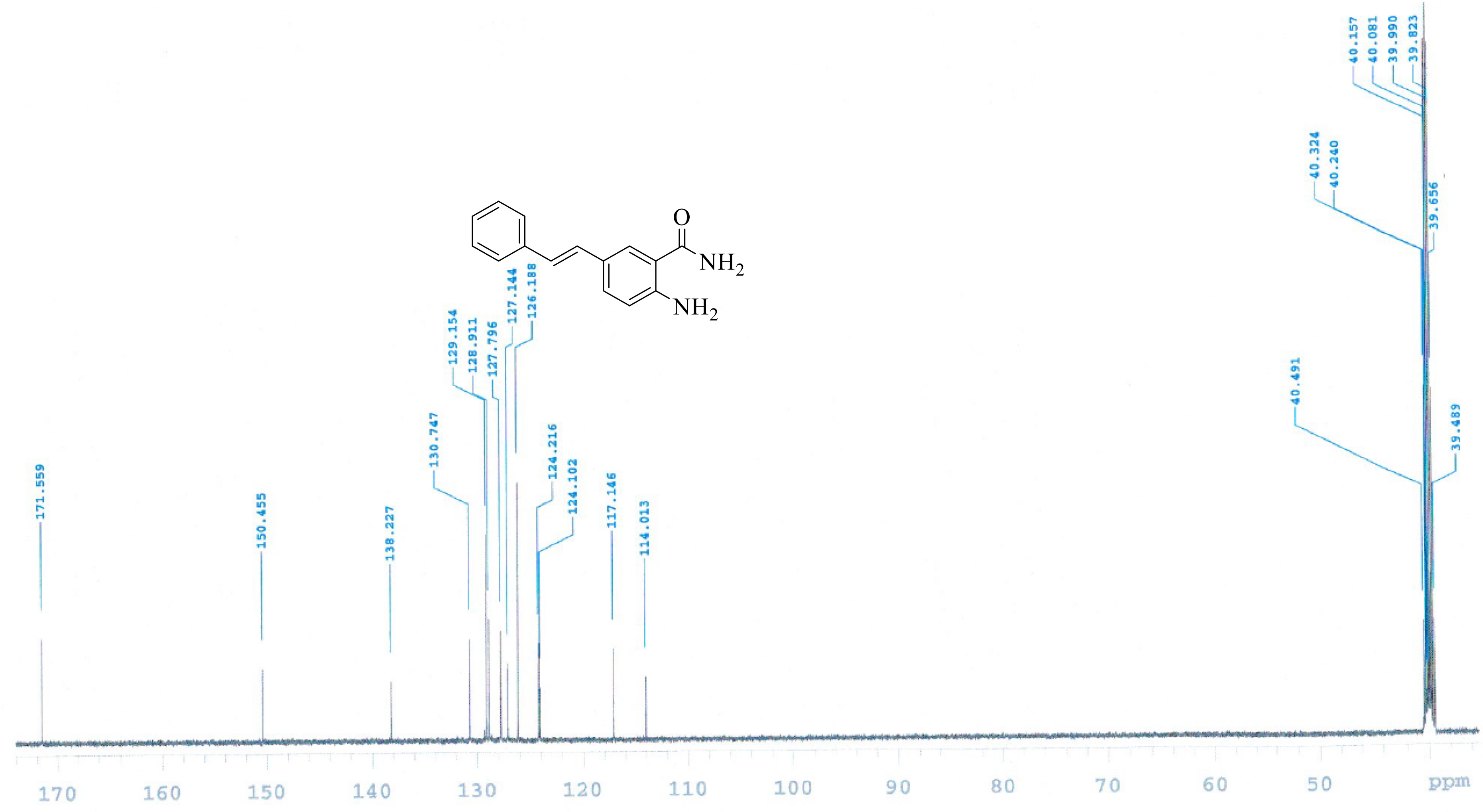

(b)

Figure S1.1: ^{1}H- and ^{13}C-NMR of **2a** in DMSO-*d*6 at 500 MHz (a) and 125 MHz (b), respectively.

(a)

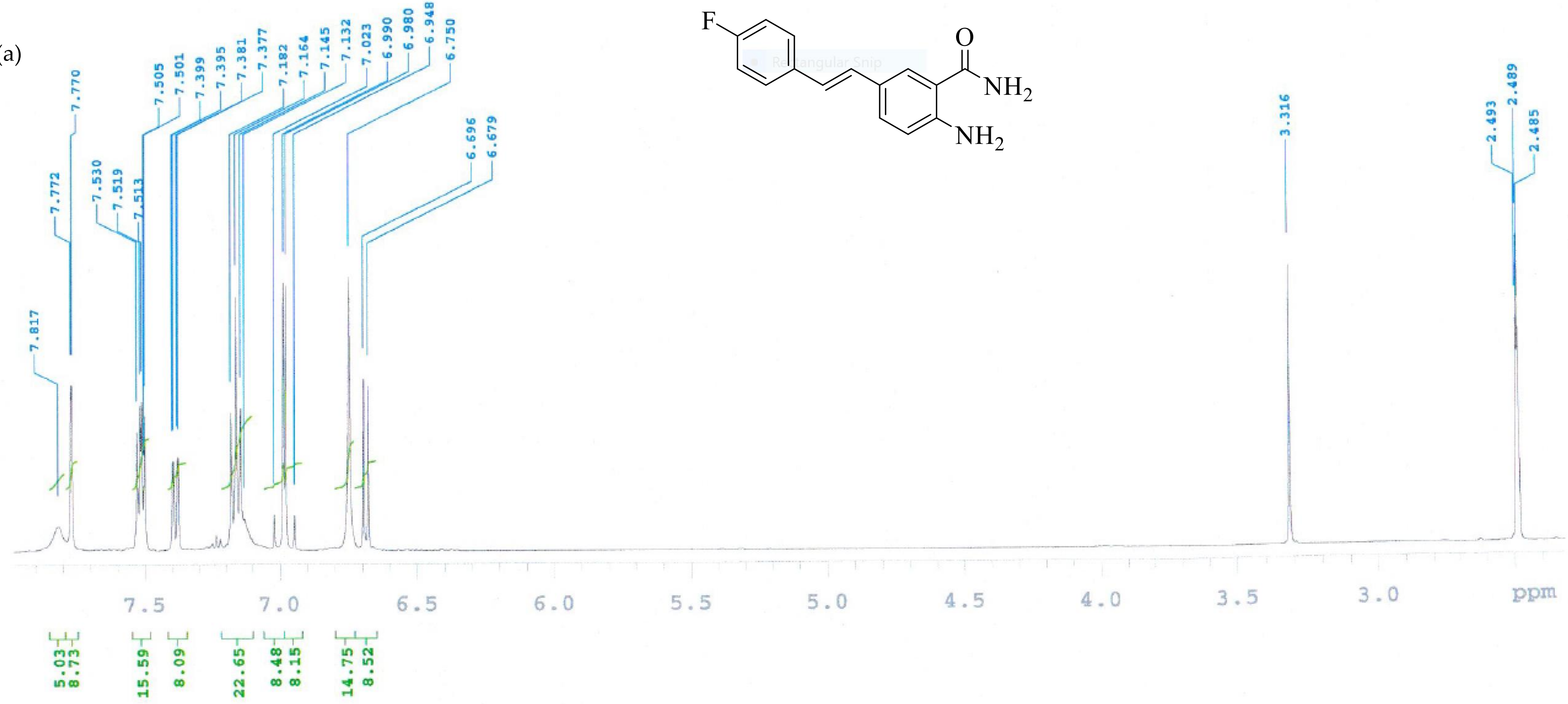

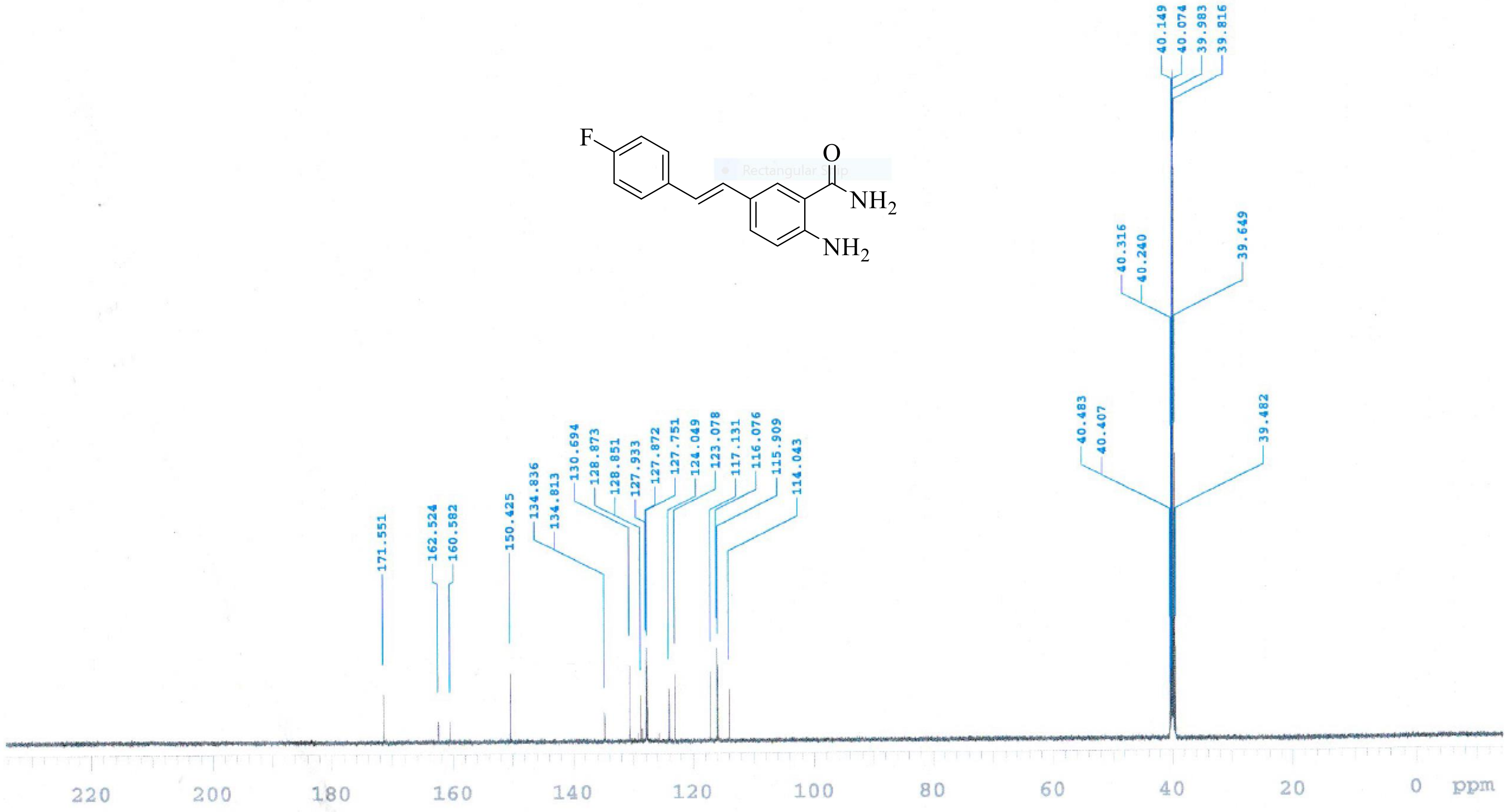

(b)

Figure S1.2: ^{1}H- and ^{13}C-NMR of **2b** in DMSO-d_6 at 500 MHz (a) and 125 MHz (b), respectively.

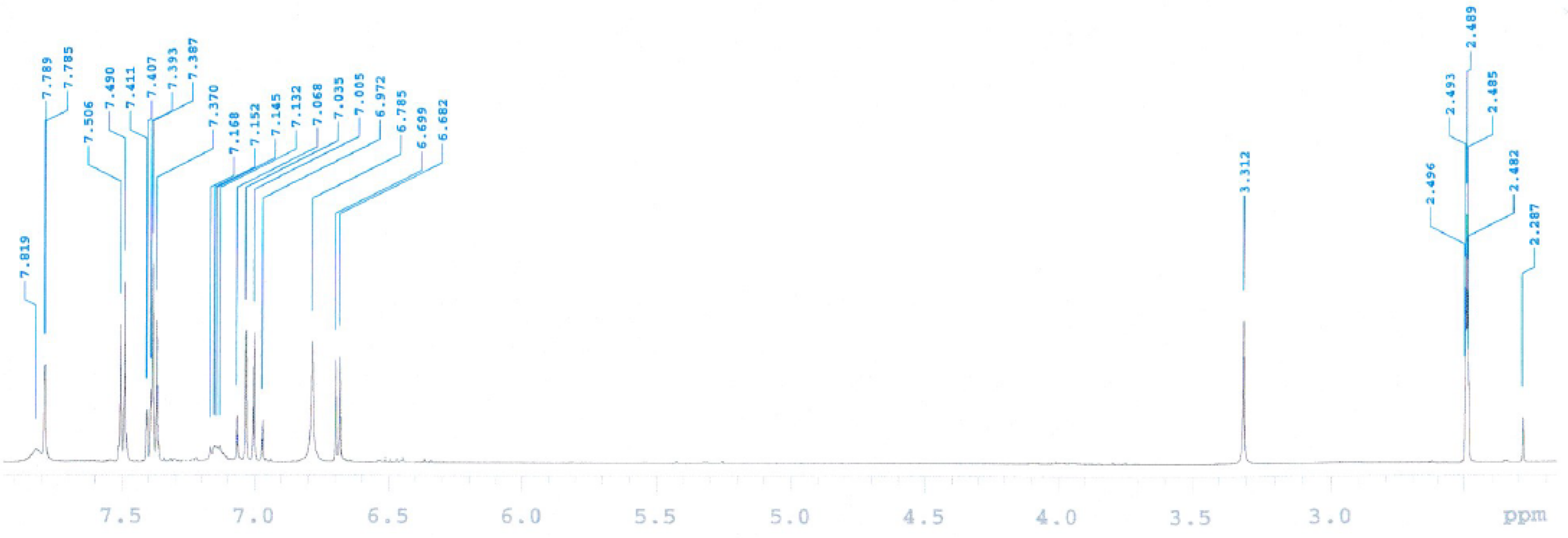
7.819
7.789
7.785
7.506
7.490
7.411
7.407
7.393
7.387
7.370
7.168
7.152
7.145
7.132
7.068
7.035
7.005
6.972
6.785
6.699
6.682
3.312
2.496
2.493
2.489
2.485
2.482
2.287
7.5
7.0
6.5
6.0
5.5
5.0
4.5
4.0
3.5
3.0
ppm

(a)

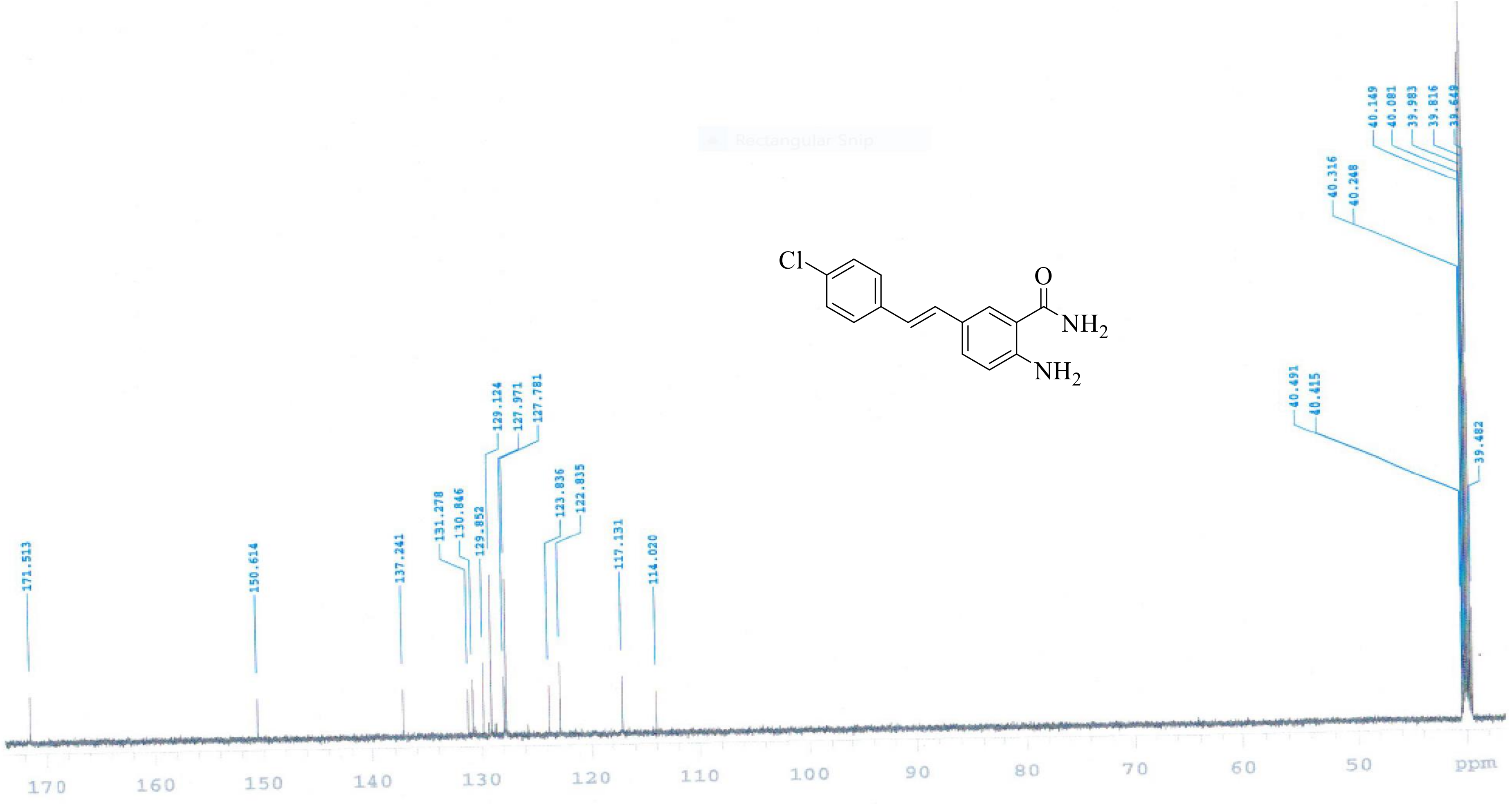

(b)

Figure S1.3: ^{1}H- and ^{13}C-NMR of **2c** in DMSO-d_6 at 500 MHz (a) and 125 MHz (b), respectively.

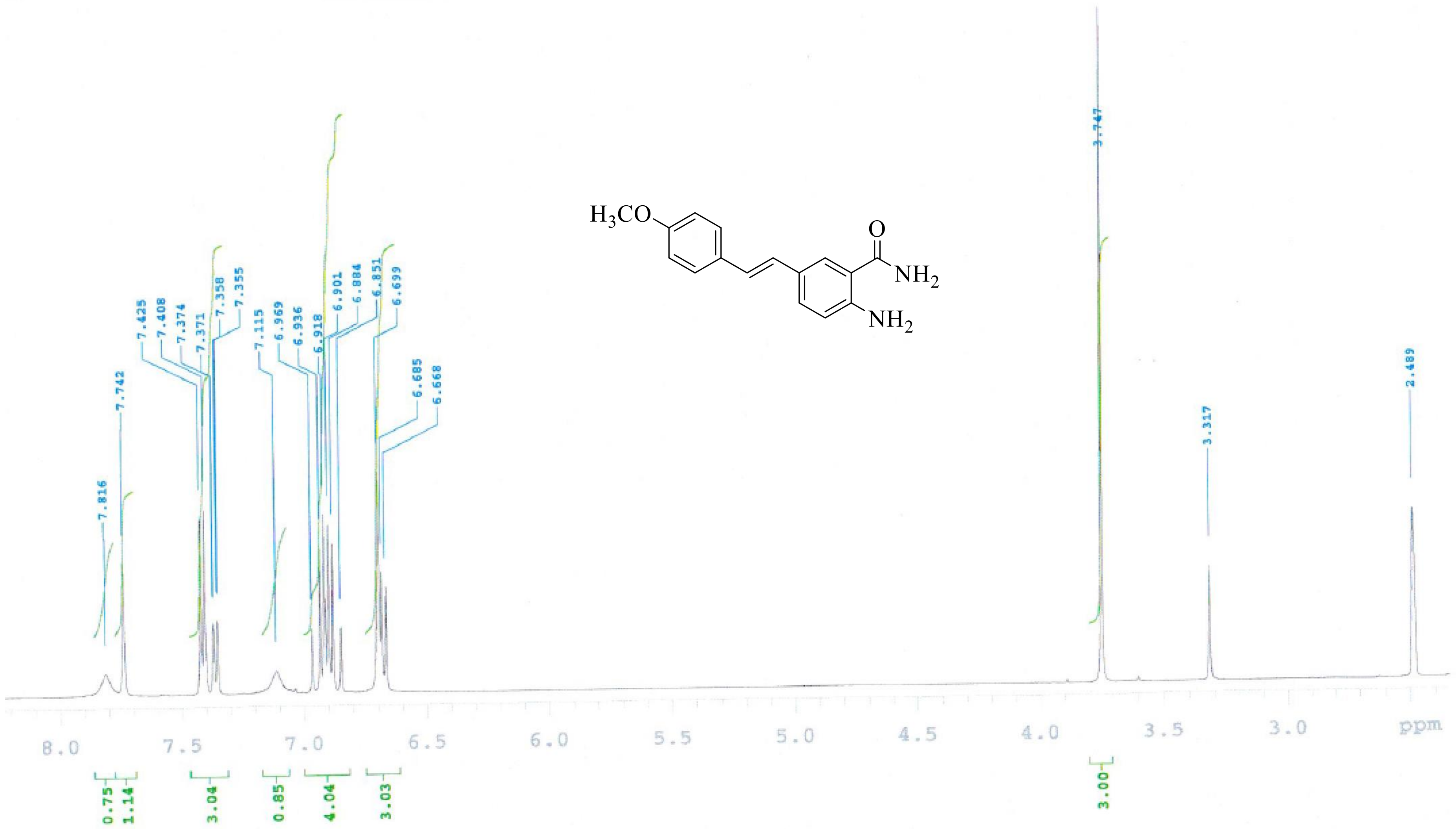
H3CO
O
NH2
NH2
7.816
7.742
7.425
7.408
7.374
7.371
7.358
7.355
7.115
6.969
6.936
6.918
6.901
6.884
6.851
6.699
6.685
6.668
3.747
3.317
2.489
8.0
7.5
7.0
6.5
6.0
5.5
5.0
4.5
4.0
3.5
3.0
ppm
0.75
1.14
3.04
0.85
4.04
3.03
3.00

(a)

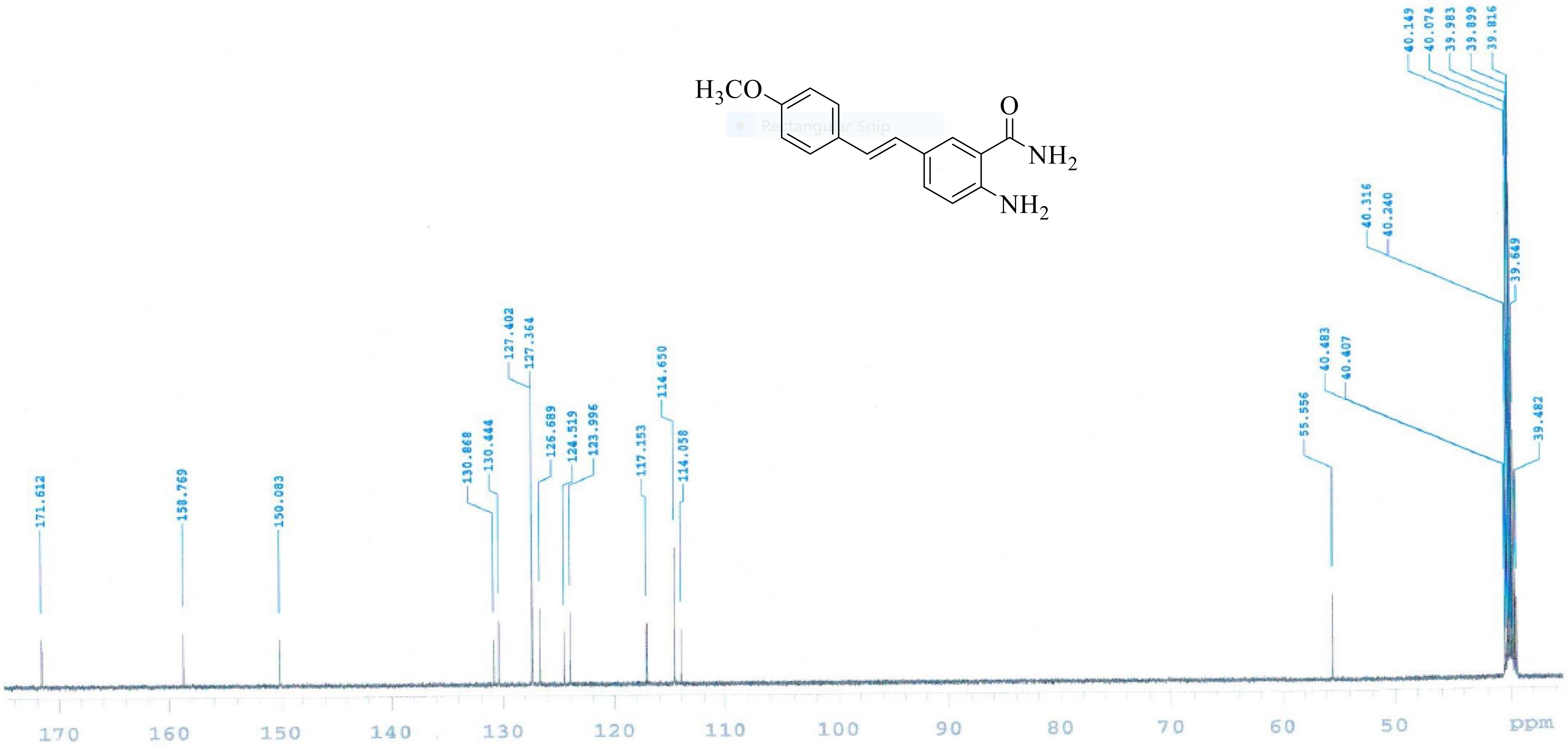

(b)

Figure S1.4: ^{1}H- and ^{13}C-NMR of **2d** in DMSO-d_6 at 500 MHz (a) and 125 MHz (b), respectively.

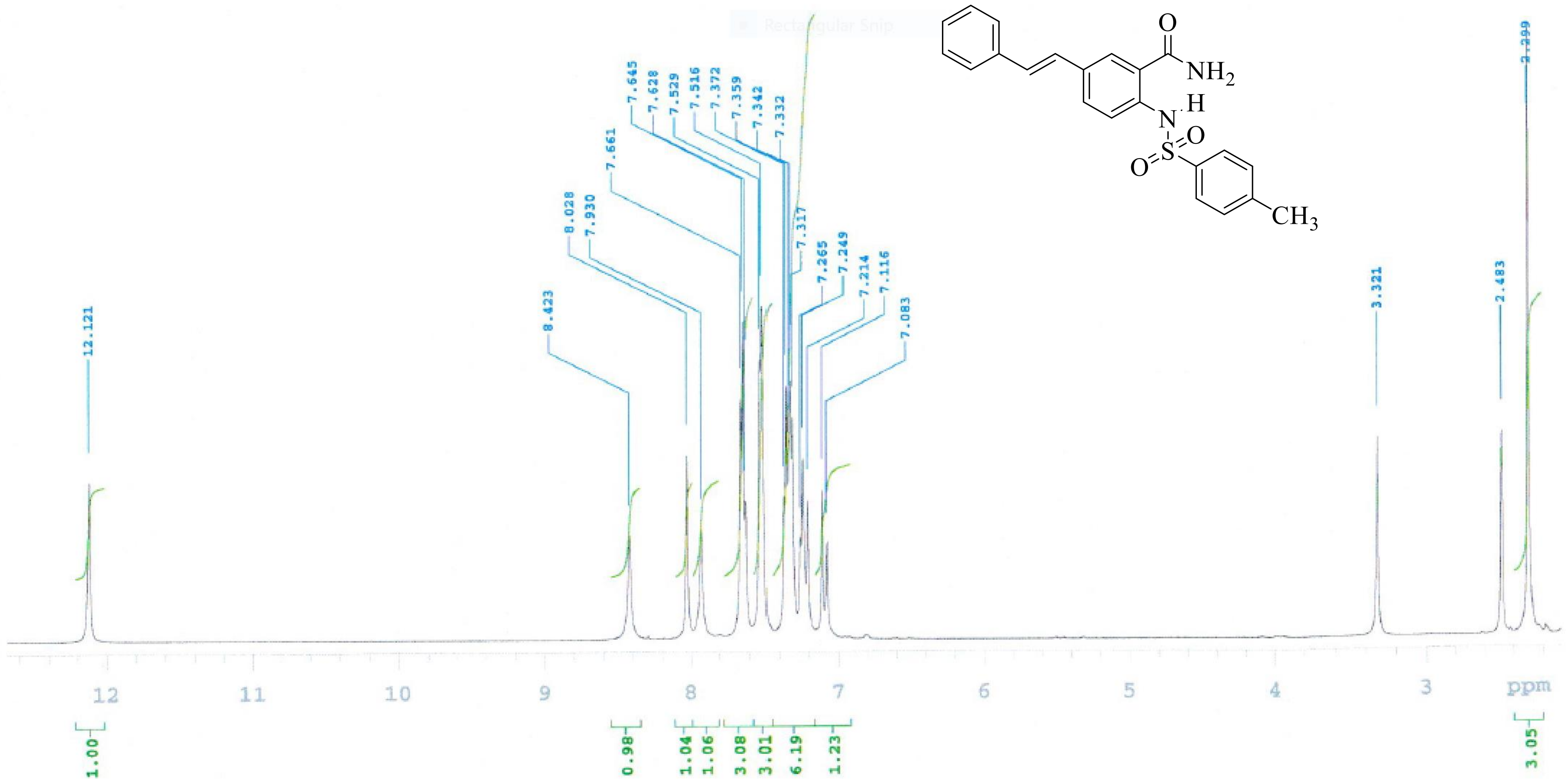

12.121
8.423
8.028
7.930
7.661
7.645
7.628
7.529
7.516
7.372
7.359
7.342
7.332
7.317
7.265
7.249
7.214
7.116
7.083
3.321
2.483
2.299
O
NH2
N
H
S
O
O
CH3
12
11
10
9
8
7
6
5
4
3
ppm
1.00
0.98
1.04
1.06
3.08
3.01
6.19
1.23
3.05

(a)

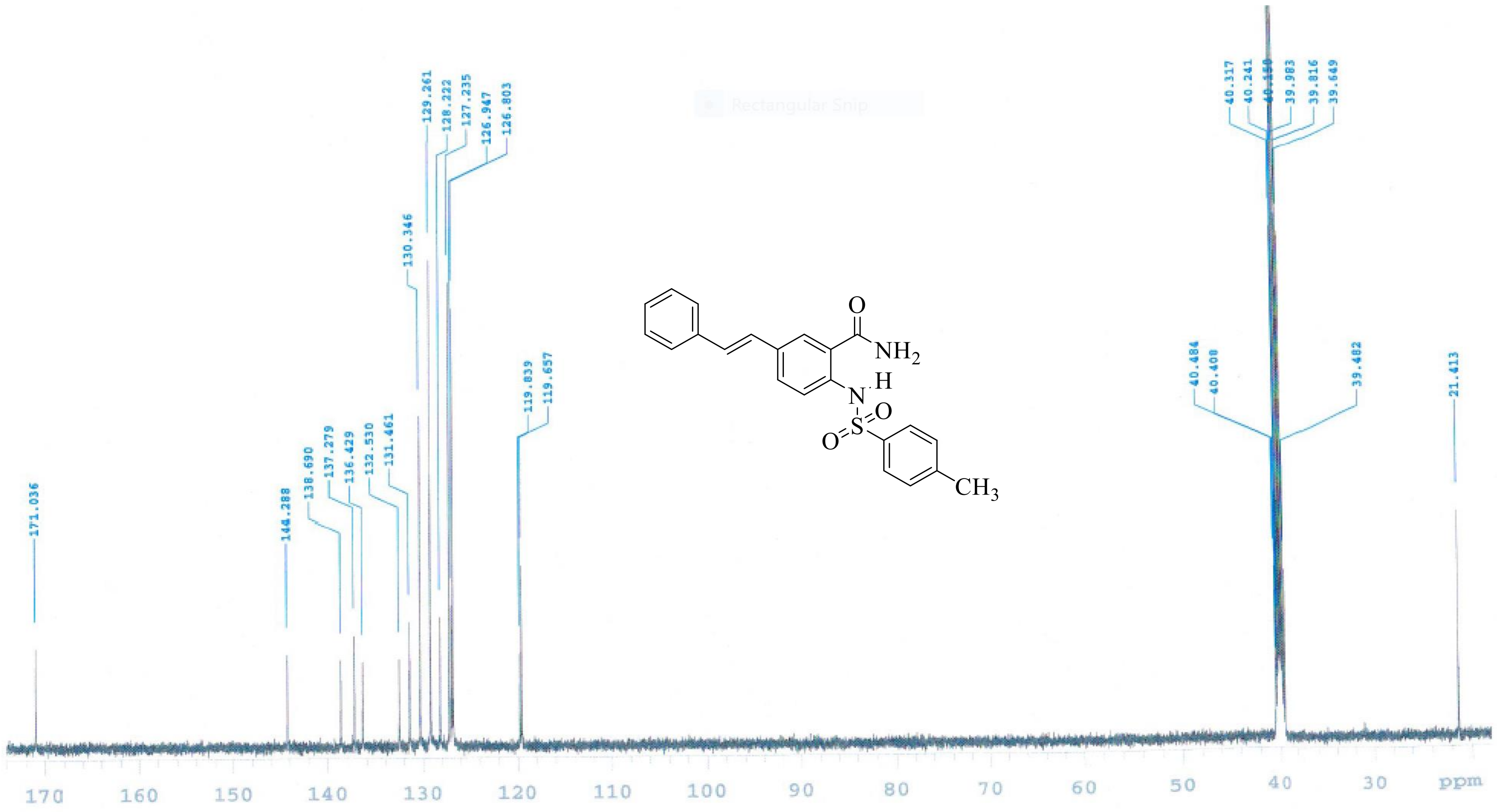

(b)

Figure S1.5: ^{1}H- and ^{13}C-NMR of **3a** in DMSO-*d*6 at 500 MHz and 125 MHz, respectively.

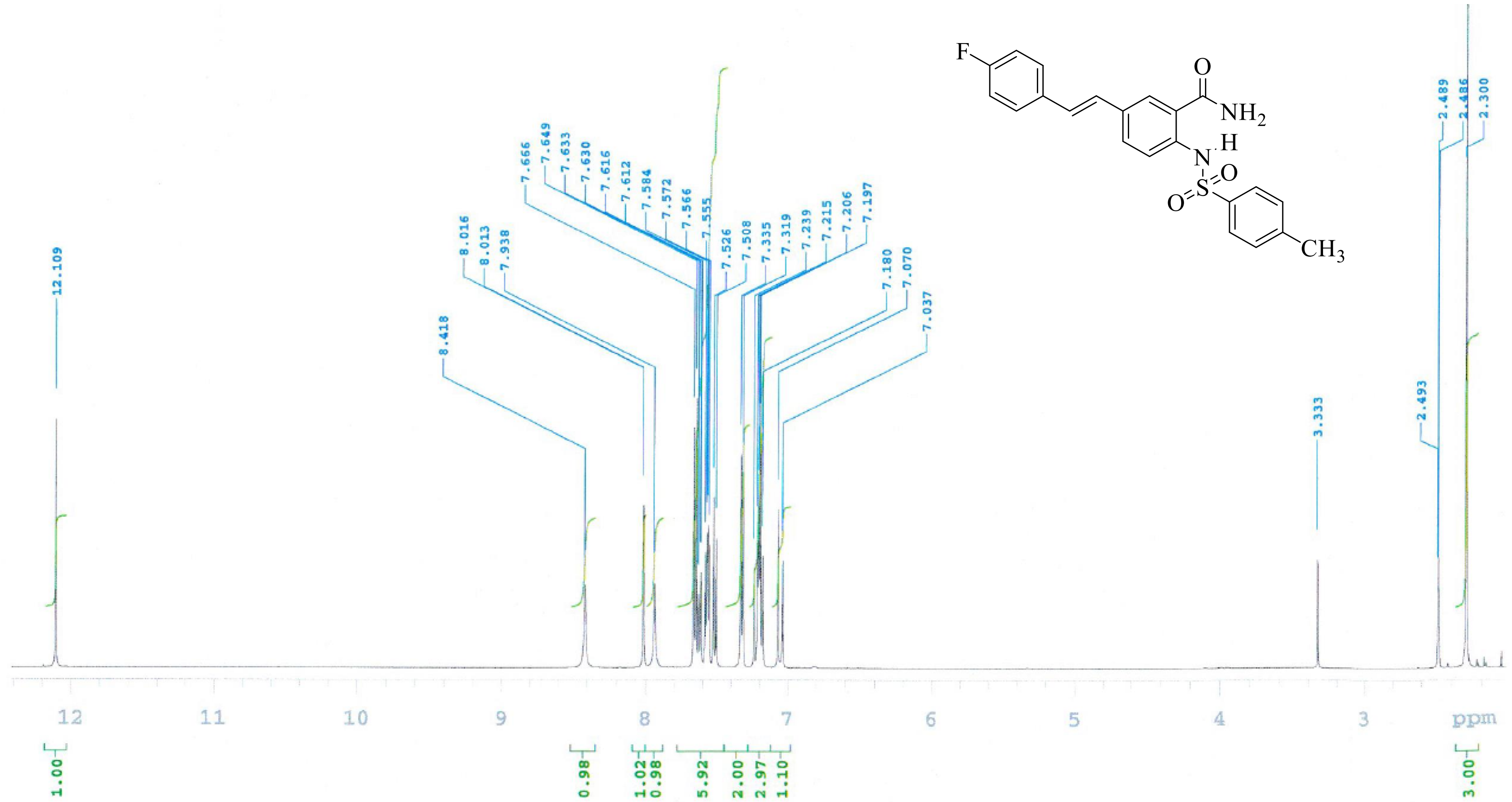
12.109
8.418
8.016
8.013
7.938
7.666
7.649
7.633
7.630
7.616
7.612
7.584
7.572
7.566
7.555
7.526
7.508
7.335
7.319
7.239
7.215
7.206
7.197
7.180
7.070
7.037
3.333
2.493
2.489
2.486
2.300
12
11
10
9
8
7
6
5
4
3
ppm
1.00
0.98
1.02
0.98
5.92
2.00
2.97
1.10
3.00
F
O
NH_2
N
H
S
O
O
CH_3

(a)

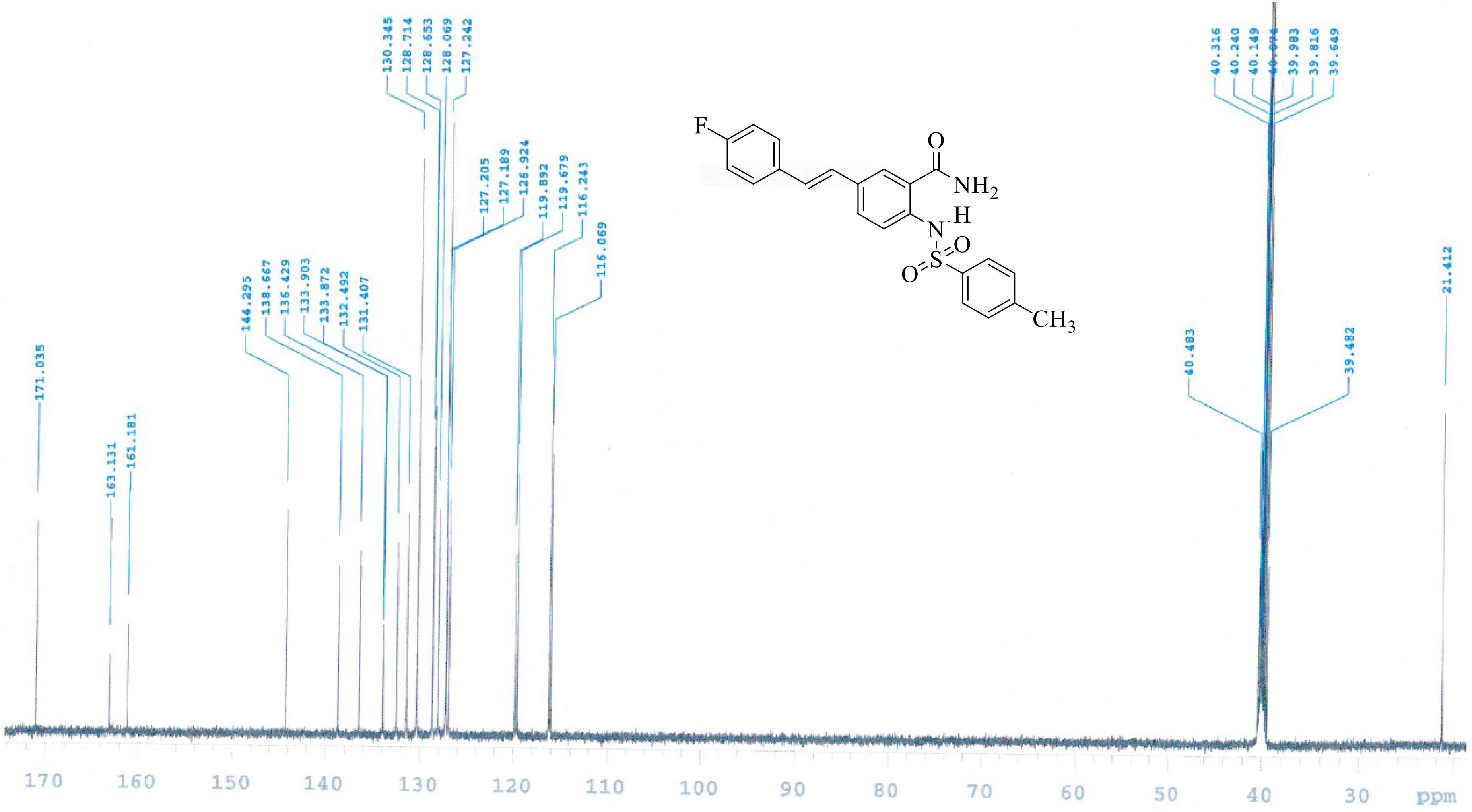

(b)

Figure S1.6: ^{1}H- and ^{13}C-NMR of **3b** in DMSO-*d*6 at 500 MHz (a) and 125 MHz (b), respectively.

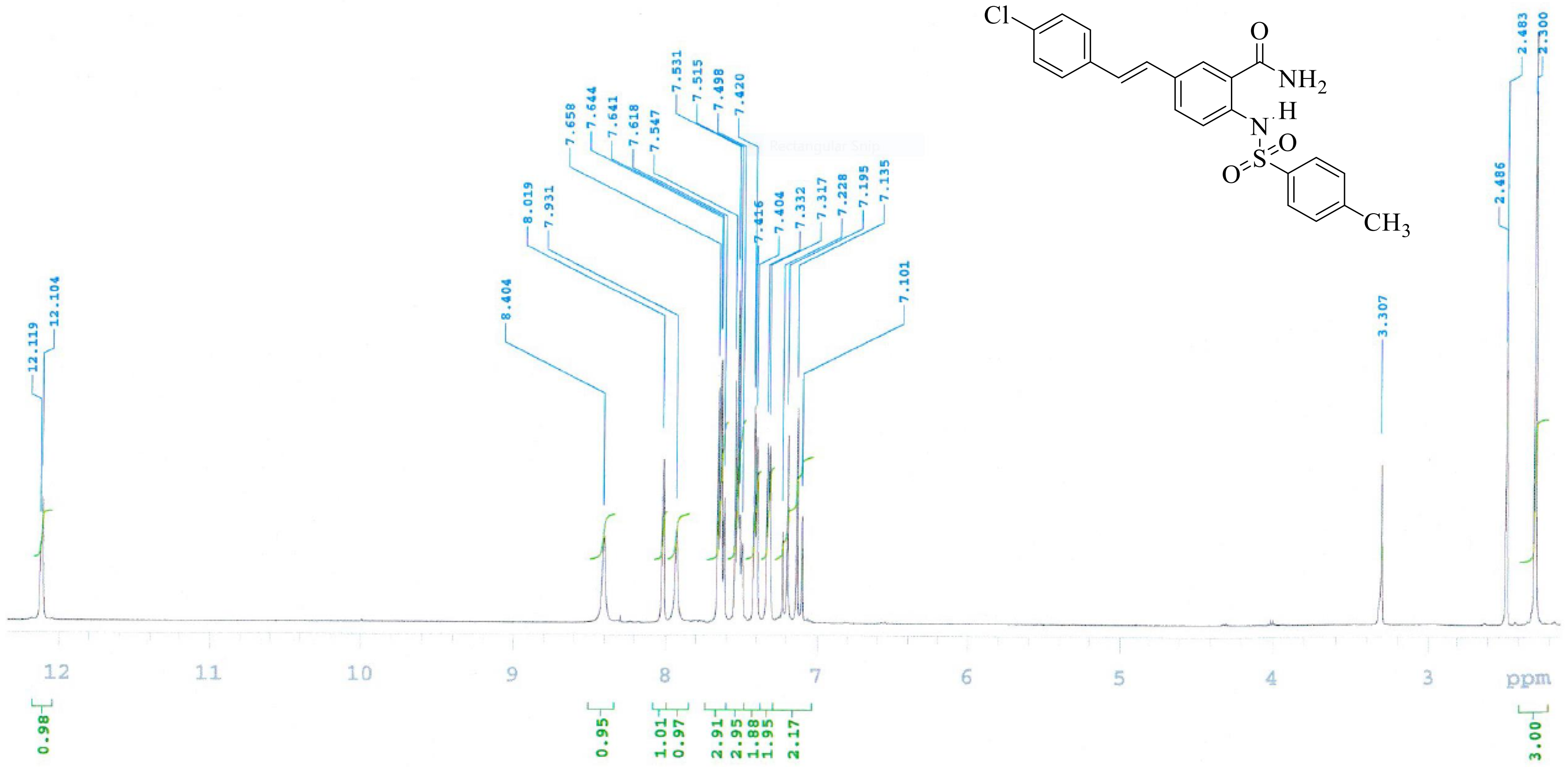

Cl
O
NH2
N
H
S
O
O
CH3
12.119
12.104
8.404
8.019
7.931
7.658
7.644
7.641
7.618
7.547
7.531
7.515
7.498
7.420
7.416
7.404
7.332
7.317
7.228
7.195
7.135
7.101
3.307
2.486
2.483
2.300
12
11
10
9
8
7
6
5
4
3
ppm
0.98
0.95
1.01
0.97
2.91
2.95
1.88
1.95
2.17
3.00

(a)

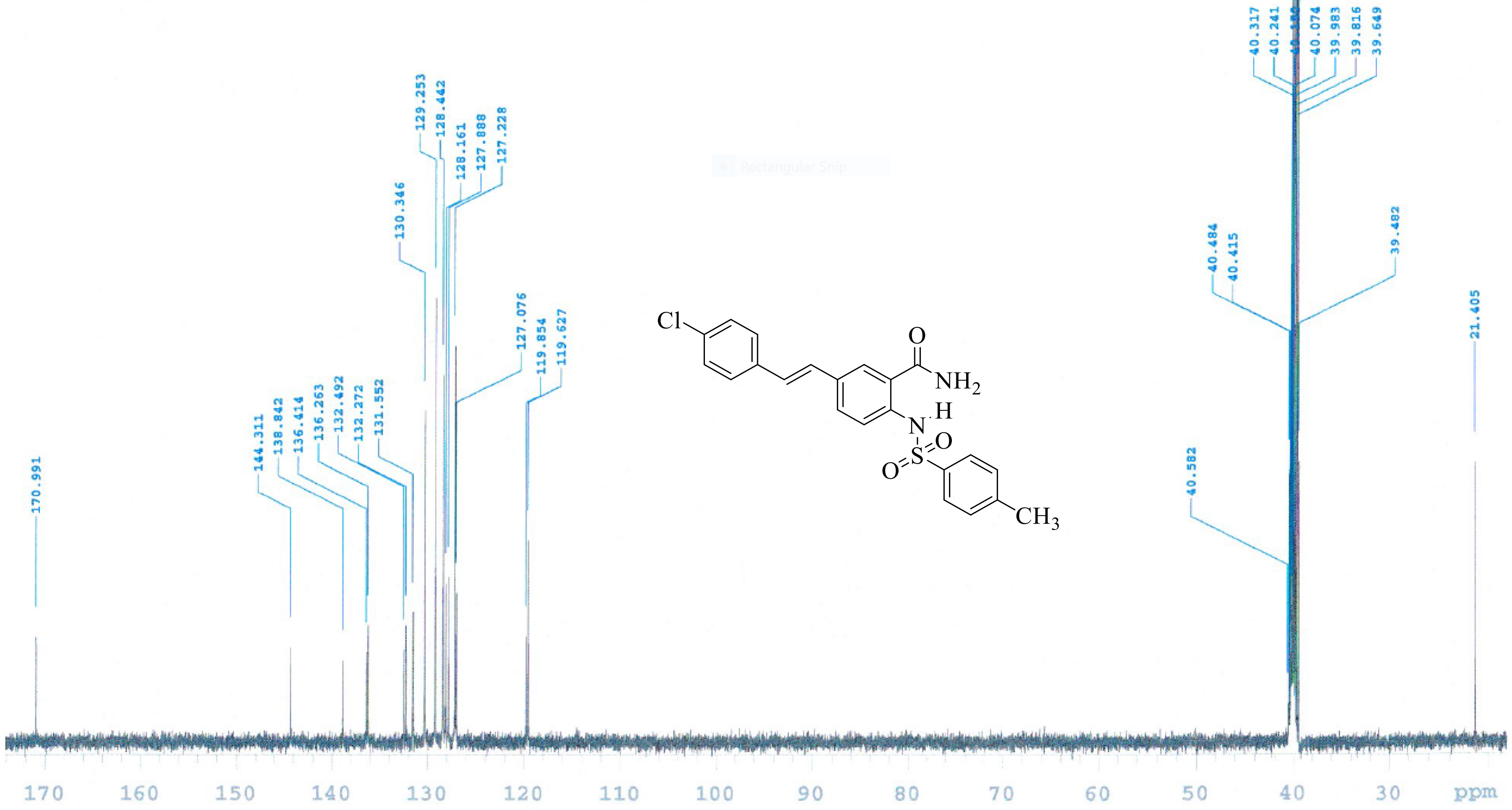

(b)

Figure S1.7: ^{1}H- and ^{13}C-NMR of **3c** in DMSO-d_6 at 500 MHz (a) and 125 MHz (b), respectively

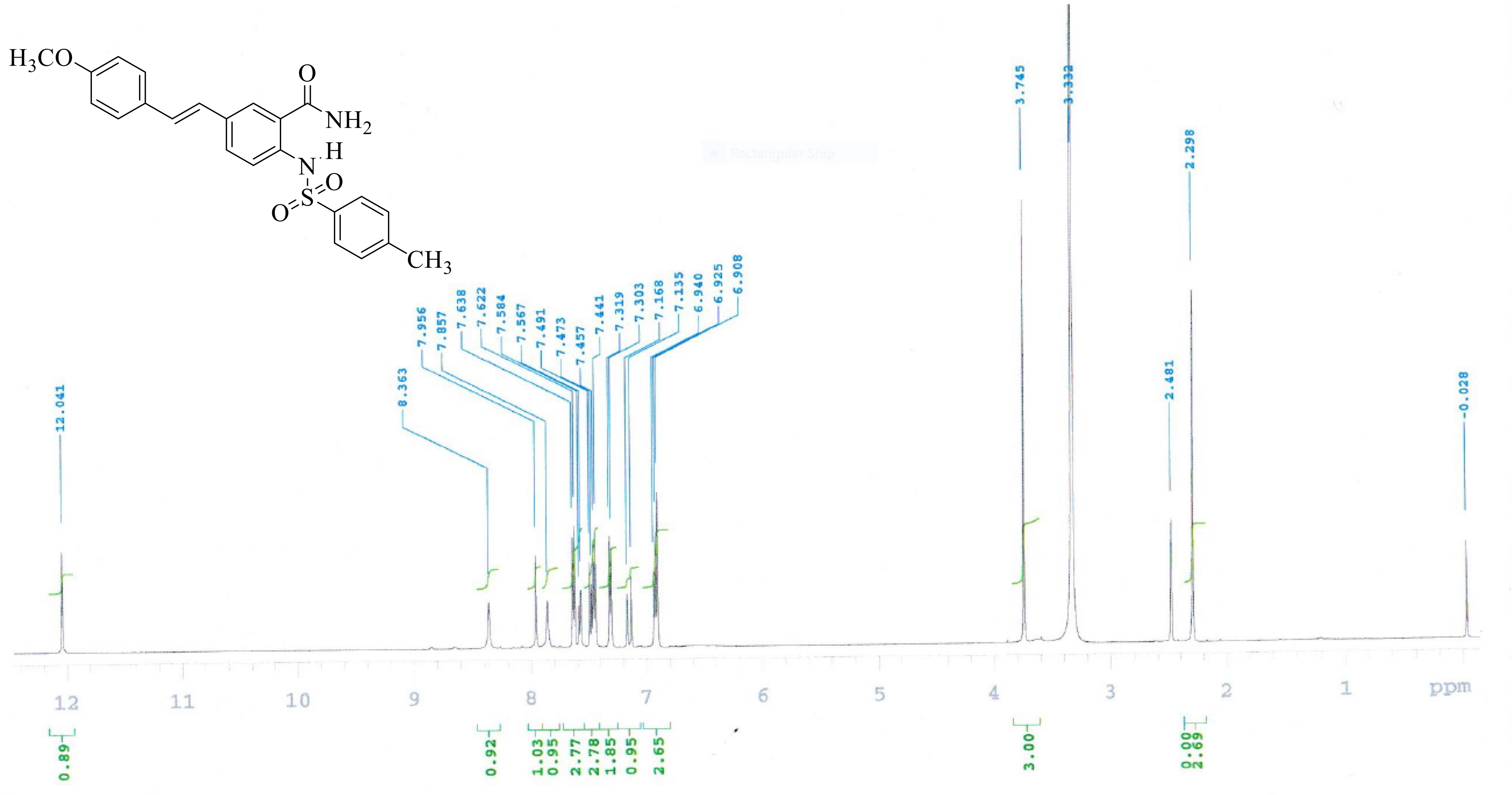
H3CO
O
NH2
N
H
S
O
O
CH3
12.041
8.363
7.956
7.857
7.638
7.622
7.584
7.567
7.491
7.473
7.457
7.441
7.319
7.303
7.168
7.135
6.940
6.925
6.908
3.745
3.332
2.481
2.298
-0.028
12
11
10
9
8
7
6
5
4
3
2
1
ppm
0.89
0.92
1.03
0.95
2.77
2.78
1.85
0.95
2.65
3.00
0.00
2.69

(a)

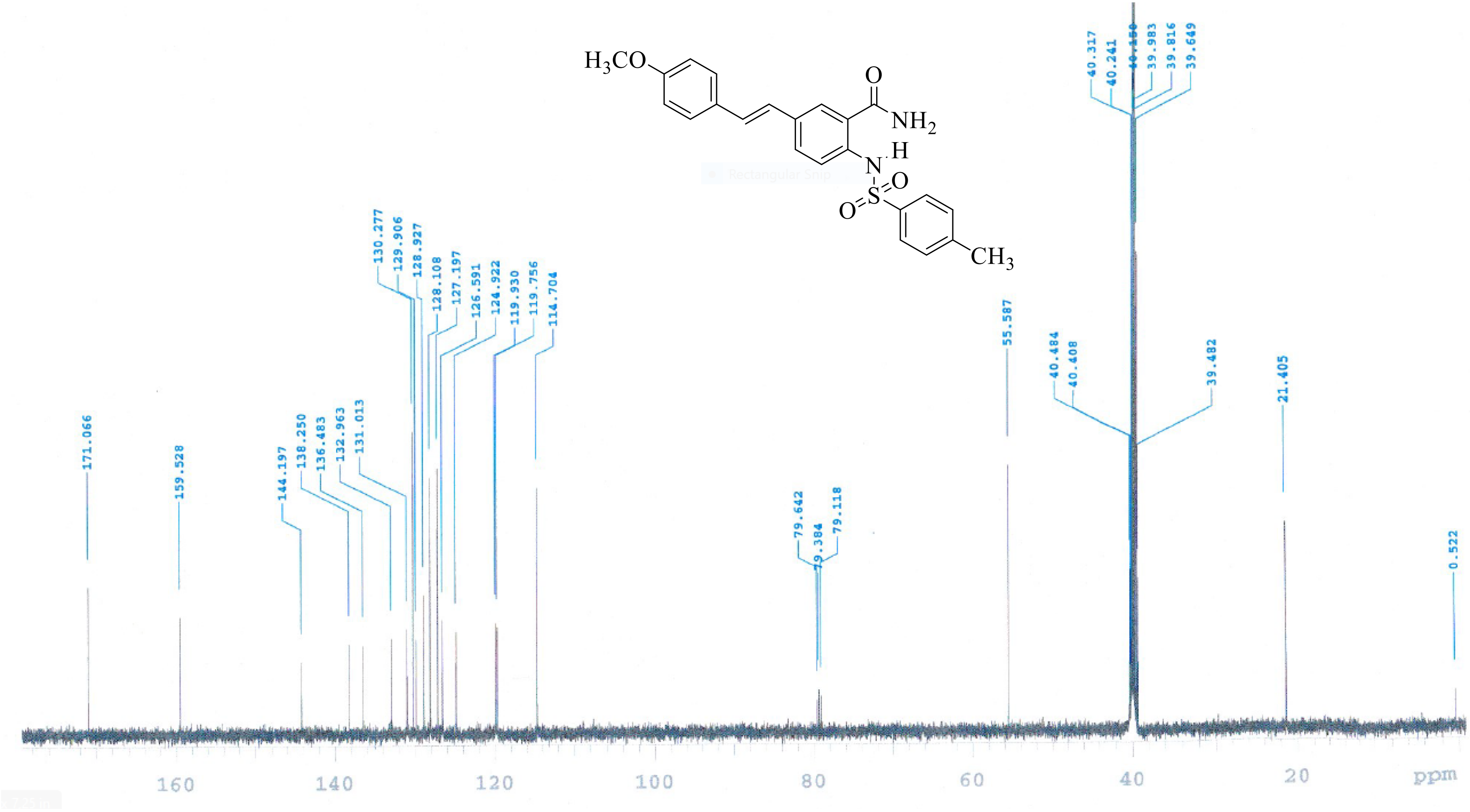

(b)

Figure S1.8: ^{1}H- and ^{13}C-NMR of **3d** in DMSO-d_6 at 500 MHz (a) and 125 MHz (b), respectively.

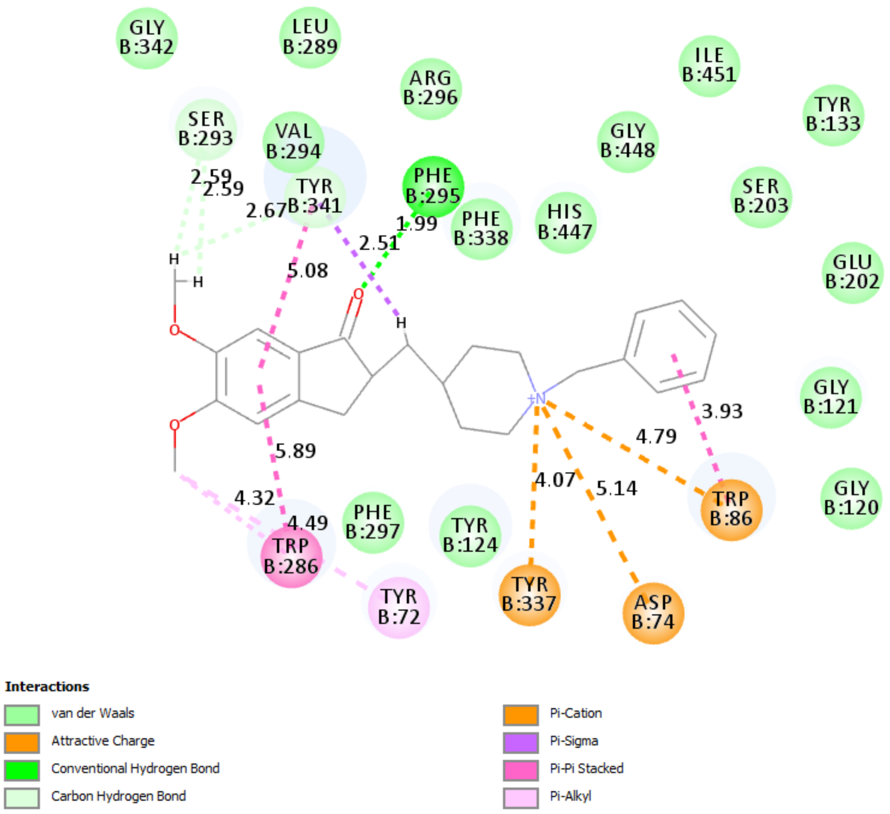
GLY B:342
LEU B:289
ARG B:296
ILE B:451
TYR B:133
SER B:293
VAL B:294
GLY B:448
PHE B:295
TYR B:341
SER B:203
PHE B:338
HIS B:447
GLU B:202
GLY B:121
GLY B:120
TRP B:86
PHE B:297
TYR B:124
TRP B:286
TYR B:72
TYR B:337
ASP B:74
2.59
2.59
2.67
1.99
2.51
5.08
3.93
4.79
5.89
4.07
5.14
4.32
4.49
Interactions
van der Waals
Attractive Charge
Conventional Hydrogen Bond
Carbon Hydrogen Bond
Pi-Cation
Pi-Sigma
Pi-Pi Stacked
Pi-Alkyl

(a)

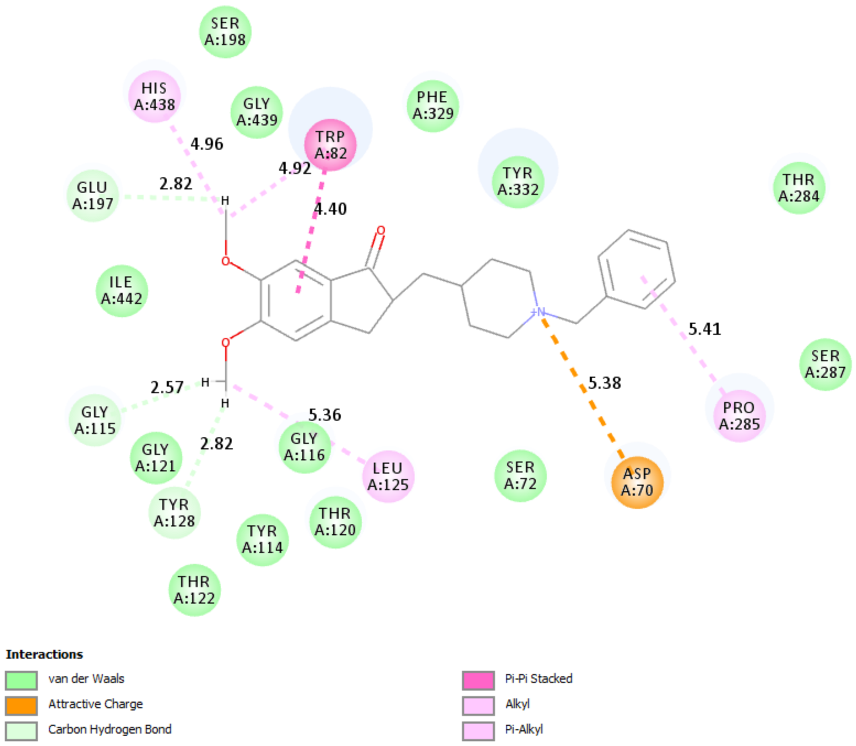
SER A:198
HIS A:438
GLY A:439
TRP A:82
PHE A:329
TYR A:332
THR A:284
GLU A:197
ILE A:442
SER A:287
PRO A:285
GLY A:115
GLY A:121
GLY A:116
LEU A:125
SER A:72
ASP A:70
TYR A:128
TYR A:114
THR A:120
THR A:122
4.96
4.92
2.82
4.40
5.41
5.38
2.57
5.36
2.82
Interactions
van der Waals
Attractive Charge
Carbon Hydrogen Bond
Pi-Pi Stacked
Alkyl
Pi-Alkyl

(b)

Figure S2: The 2-dimensional (2D) plots of docking results for donepezil into AChE (**a**) and BChE (**b**), including bonding interaction distances and colour codes for various interactions.
